# Parkinson's Disease: A Comprehensive Analysis of Fungi and Bacteria in Brain Tissue

**DOI:** 10.7150/ijbs.42257

**Published:** 2020-02-10

**Authors:** Diana Pisa, Ruth Alonso, Luis Carrasco

**Affiliations:** Centro de Biología Molecular “Severo Ochoa” (CSIC-UAM). c/Nicolás Cabrera, 1. Universidad Autónoma de Madrid. Cantoblanco. 28049 Madrid. Spain.

**Keywords:** Parkinson's disease, neurodegenerative diseases, polymicrobial infections, fungal infection, next-generation sequencing

## Abstract

Parkinson's disease (PD) is characterized by motor disorders and the destruction of dopaminergic neurons in the substantia nigra pars compacta. In addition to motor disability, many patients with PD present a spectrum of clinical symptoms, including cognitive decline, psychiatric alterations, loss of smell and bladder dysfunction, among others. Neuroinflammation is one of the most salient features of PD, but the nature of the trigger remains unknown. A plausible mechanism to explain inflammation and the range of clinical symptoms in these patients is the presence of systemic microbial infection. Accordingly, the present study provides extensive evidence for the existence of mixed microbial infections in the central nervous system (CNS) of patients with PD. Assessment of CNS sections by immunohistochemistry using specific antibodies revealed the presence of both fungi and bacteria. Moreover, different regions of the CNS were positive for a variety of microbial morphologies, suggesting infection by a number of microorganisms. Identification of specific fungal and bacterial species in different CNS regions from six PD patients was accomplished using nested PCR analysis and next-generation sequencing, providing compelling evidence of polymicrobial infections in the CNS of PD. Most of the fungal species identified belong to the genera *Botrytis, Candida, Fusarium and Malassezia*. Some relevant bacterial genera were *Streptococcus* and *Pseudomonas*, with most bacterial species belonging to the phyla *Actinobacteria* and *Proteobacteria*. Interestingly, we noted similarities and differences between the microbiota present in the CNS of patients with PD and that in other neurodegenerative diseases. Overall, our observations lend strong support to the concept that mixed microbial infections contribute to or are a risk factor for the neuropathology of PD. Importantly, these results provide the basis for effective treatments of this disease using already approved and safe antimicrobial therapeutics.

## Introduction

One of the most important challenges in modern medicine is the elucidation of the etiology of neurodegenerative diseases such as Alzheimer's disease (AD), Parkinson's disease (PD), amyotrophic lateral sclerosis (ALS) and multiple sclerosis (MS), each of which has remained obscure. PD is a progressive and debilitating disease that induces motor disorders, rigidity and dyskinesia through the functional loss of dopaminergic neurons in the substantia nigra 1. PD affects over 6 million people worldwide 2-4, with the vast majority of patients diagnosed in their sixth decade of life. In addition to movement disorders, many patients show other clinical symptoms, including psycho emotional and cognitive deficits, dementia, sleep disturbances and olfactory and bladder dysfunction, among others 5-9. Some of these symptoms can present years before diagnosis, prior to when motor signs are apparent. Progressive cognitive decline has been associated with gray matter atrophy, cortical thinning and considerable alterations to both gray and white matter 10. Moreover, global brain atrophy and modifications in several CNS regions are present in addition to the destruction of the substantia nigra, including the amygdala, thalamus and right caudate hippocampus and nucleus accumbens 11, 12. In fact, PD represents a very heterogeneous disease and each patient differs regarding their symptoms and clinical progression and, accordingly, several subtypes of PD have been proposed 13. It has been suggested that the initiation of neurodegeneration in PD occurs in the olfactory bulb, which in some instances reduces its volume by about 50% 14-16. Thus far, treatments for PD are directed to alleviate clinical symptoms, for example, by maintaining the levels of dopamine in the striatum 17, 18. There are no treatments to stop or reverse the course of the disease.

An important neuropathological hallmark of PD is the abnormal deposits of synuclein in some neurons of several brain regions 4, 14. These aggregates were described as Lewy bodies over a century ago, and initially appear in cholinergic and monoaminergic brainstem neurons in the olfactory system, but are also found in limbic and neocortical brain regions as the disease progresses 19. Other cellular functions are also affected, such as mitochondrial activity, oxidative stress, calcium homeostasis and axonal transport. Another important pathological feature of PD is systemic inflammation 20-22. Neuroinflammation is likely an essential contributor to PD pathogenesis of the CNS, and the elevation in the levels of a number of cytokines and interleukins in peripheral blood of patients point to the stimulation of the immune system 23. The presence of inflammation has prompted the suggestion that PD is caused by viral or bacterial infection. Thus, it has been speculated that influenza virus or *Actinobacteria* may be involved in the etiology of PD 24-27. It has been suggested that molecular mimicry between HSV1 and α-synuclein could foster the progression of PD 28, 29. Also, the possibility that some toxins or fungi can provoke PD has been suggested 30-34. More recently, the possibility that Malassezia might be contributing to PD has been hypothesized 35. However, the direct demonstration that these pathogens are present in brain tissue of PD patients was not provided. A possible link between the gut microbiota and neurodegeneration has also been developed in recent years 36-38. Thus, the gut microbiota can influence CNS functioning, microglia activation and, in some instances, may lead to the synthesis of metabolites that provoke the pathological communication of gut microbes with microglia in the CNS and induce degeneration by the synthesis of toxic molecules. In the context of PD, the gut microbiota differs from control individuals and can influence α-synuclein aggregation 37, 39, 40. Accordingly, the gut microbiota in each PD patient may contribute to disease pathogenesis 36, 41. With respect to fungal infection, a connection between the mycobiota and neurodegeneration has been recently reviewed 41.

Similarities between PD and other neurodegenerative diseases have been noted both in clinical symptoms and in cytoplasmic protein aggregates 42-44. Specifically, PD and AD share cognitive decline, the formation of amyloid plaques and phosphorylated tau and α-synuclein aggregates in the cytoplasm of neurons 45, 46. Hence, it is possible that both diseases may share a similar pathological agent that affects different CNS regions. For some years, we have advanced the concept that these diseases might be caused by polymicrobial infections 47-52, involving a variety of fungal and bacterial species that can progressively colonize the central nervous system (CNS) 47, 51, 53, 54. Some of these microbial cells can be found inside neurons and are located intranuclearly. In addition, *corpora amylacea* (CA) from both AD or PD patients contain fungal components, supporting the concept that this infection is located at the CNS, and that these bodies have formed during months or even years, trapping microbial antigens in the brain 55. Against this background, the aim of the present study was to investigate the presence of both fungi and bacteria in brain tissue from patients with PD. Our results provide direct evidence of the existence of both fungi and bacteria with different morphologies in neural tissue from several CNS regions. The precise fungal and bacterial species found in each PD patient was determined using nested PCR and next-generation sequencing (NGS). Collectively, our present findings provide the first direct evidence of fungal and bacterial infection of PD brains.

## Materials and Methods

### CNS samples from PD patients

We obtained CNS sections and frozen tissue from six patients diagnosed with PD, in addition to samples from four CNS regions of four control subjects. The age and gender of each patient and control subject are listed in Table S1. The *Banco de Tejidos CIEN*, Madrid, supplied the samples, which were analyzed anonymously. Sample transfer was carried out according to national regulations concerning research on human biological samples and written informed consent was obtained in all cases. This study was approved by the ethics committee of the Universidad Autónoma de Madrid. To avoid contamination, frozen tissue was handled with sterile instruments in a laminar flow hood.

### Antibodies employed

A number of rabbit polyclonal antibodies used in this study have been previously described (Pisa et al., 2015; Pisa et al., 2016a). These antibodies were raised against *Candida albicans* (used at 1:100 dilution), *C. glabrata* (used at 1:500 dilution), *Syncephalastrum racemosum* (used at 1:100 dilution) and *Phoma betae* (used at 1:100 dilution). A rabbit polyclonal antibody against fungal chitin was generously provided by Dr. M.N. Horst (Mercer University, Georgia), used at 1:50 dilution. Rabbit polyclonal antibodies against purified fungal enolase and β-tubulin were produced in our laboratory and have been described 56; both were used at 1:50 dilution. Also, a rat polyclonal antibody against *Trichoderma viride* was obtained in our laboratory 47, and was used at 1:20 dilution. The remaining antibodies were purchased from several commercial vendors: mouse monoclonal antibody against human α-tubulin (Sigma-Aldrich, St Louis, MI), mouse monoclonal antibody against anti-human neurofilament protein, clone 2F11 (Dako, Carpenteria, CA), both used at 1:50 dilution; mouse monoclonal antibody against bacterial peptidoglycan (Thermo Fisher Scientific, Waltham, MA) and rabbit polyclonal antibody against *Chlamydophila pneumoniae*, which immunoreacts with the major outer porin (Biorbyt, Cambridge, UK), both used at 1:20 dilution.

### Immunohistochemistry

Standard techniques were used for paraffin embedding and sectioning of CNS tissue. Briefly, paraffin was removed and tissues were rehydrated and pretreated with different buffer solutions. Subsequently, tissue sections were incubated with a primary antibody in PBS-BSA and further incubated with the corresponding secondary antibody conjugated to Alexa 488 (Invitrogen, Carlsbad, CA). Sections were then incubated with a third antibody in PBS and further incubated with the corresponding secondary antibody conjugated to Alexa 555 (Invitrogen). The tissue sections were stained with DAPI and then with autofluorescence eliminator reagent (Merck Millipore). Protocols for immunohistochemical analysis have been described 56. Most of the images were obtained with a Zeiss LSM710 confocal laser scanning microscope equipped with the upright AxioImager.M2 stand (Zeiss), running ZEN 2010 software, or a Zeiss LSM800 confocal laser-scanning microscope equipped with an inverted microscope Axio Observer (Zeiss) and running Zen Blue 2.3 software. Images were deconvoluted using Huygens software (4.2.2 p0) and visualized with ImageJ (NIH).

### DNA extraction from CNS tissue

DNA was extracted using the QIAmp (Qiagen) genomic DNA isolation kit from frozen CNS tissue of six patients and four control subjects. The CNS regions examined corresponded to: motor cortex (MC), medulla (MD), mesencephalon (MS), hypothalamus (HT), pons (PN), callosal body (CB), caudate and lenticular nuclei (CLN), lateral frontal cortex (LFC), entorhinal (ERH) and spinal cord (SC). Samples were treated with different buffer solutions and incubated at 56ºC and at 70ºC for 2 hours and 10 min, respectively (Alonso et al 2017a). Subsequently, samples were applied to the QIamp Mini spin column and centrifuged. Finally, each sample was collected in distilled water.

### Nested PCR

To prevent PCR contamination, we used separate rooms and specific glassware supplies for PCR set-up and products, aliquoted reagents, positive-displacement pipettes, aerosol-resistant tips and several negative controls. Different sets of primers were used to amplify the internal transcribed spacer (ITS1 and ITS2) regions of fungal ribosomal DNA. Primer design for PCR amplification has been previously described in detail 57. First, we amplified the ITS1-region: the first PCR was carried out using 2 μl of DNA incubated at 95°C for 10 min, followed by 30 cycles of 45 s at 94°C, 1 min at 57.3°C and 45 s at 72°C. Primers used in the first PCR were forward and reverse ITS-1 (External 1). The second PCR was performed using 2 μl of the product obtained in the first PCR, using forward and reverse ITS-1 (Internal 1) primers for 35 cycles of 45 s at 94°C, 1 min at 55°C and 45 s at 72°C. A separate PCR assay was designed to amplify the ITS-2 region: the first PCR assay was carried out with 2 μl of DNA incubated at 95°C for 10 min, followed by 30 cycles of 45 s at 94°C, 1 min at 52°C and 45 s at 72°C. Oligonucleotides used in the first PCR were forward and reverse ITS-2 (External 2). The second PCR was carried out with 2 μl of the product obtained in the first PCR and forward and reverse ITS-2 (Internal 2) primers, for 30 cycles of 45 s at 94ºC, 1 min at 55ºC, and 45 s at 72ºC.

To assay for bacterial DNA, we amplified the intergenic spacer (IGS) region between rRNA genes of the prokaryotic genome using the primers 1406(F) / 559(R) and 1492(F) / 242(R) in the first and second round PCR, respectively: the first PCR was carried out with 4 μl of DNA denatured at 95°C for 5 min, followed by 45 cycles of 1 min at 94°C, 1 min at 55°C and 3 min at 72°C. The second PCR was performed using 2 μl of the product obtained in the first PCR, for 35 cycles of 1 min at 94°C, 1 min at 57°C and 3 min at 72°C. Some PCR products were sequenced by Macrogen Inc. (Seoul, Korea). All sequences have been deposited into the European Nucleotide Archive with the access numbers PRJEB32342 and PRJEB32365

### Next-generation sequencing

NGS was used to characterize the ITS-region of fungi and the 16Sr DNA gene of bacteria. NGS was undertaken at the Genomics Unit of the Scientific Park of Madrid. Quality analyses were performed over reads using FastQC software (http://www.bioinformatics.babraham.ac.uk/projects/fastqc/). All sequences have been submitted to European Genome-phenome Archive with the accession number EGAS00001003643 and EGAS00001003644.

### Fungi

The region between the Internal 1 primers was amplified with specific primers joined to linker sequences in a first round of PCR (specific product of ~300 nt). A second PCR was performed on this product using fusion primers containing Illumina and linker sequences. In the case of bacteria, primers were designed to amplify the region between the variable V3-V4 region of the 16S rDNA gene. These primers were joined to linker sequences in a first round of PCR (specific product of ~400 nt). A second PCR was performed on this product using fusion primers containing Illumina and linker sequences**.** PCR products were sequenced on a MiSeq sequencing platform (Illumina).

### Computational analysis

Metagenomics results were analyzed using QUIIME 58, an open-source bioinformatics pipeline that is designed to take users from raw sequencing data generated on the Illumina or other platforms to publication-quality graphics and statistics, including quality filtering, operational taxonomic unit (OTU) picking, taxonomic assignment and phylogenetic reconstruction, and diversity analyses and visualizations. The adapters from the sequences were deleted using Cutadapt and all sequences with a length shorter than 35 bp were discarded. Once the sequence set-up was ready, we performed a metagenomic-type analysis that consisted of several steps (http://nbviewer.ipython.org/github/biocore/qiime/blob/1.9.1/examples/ipynb/Fungal-ITS-analysis.ipynb).

#### Sequence clustering

The sequences of all samples were grouped to define the OTUs using the pick_open_reference_otus.py workflow (http://qiime.org/) with a percentage identity of 97% and 94% in fungi and bacteria, respectively.

#### Identification of OTU hots from uncultured fungi

According to the taxonomical classification (species level), we found that on average 42% of the matches corresponded to “Uncultured fungus Blast” hit. For this reason an additional standard Blast search analysis was performed.

### Principal Component Analysis

We used β-diversity parameter tests for assessing diversity between samples. The core diversity analyses previously performed calculated this parameter as well using a Principal Coordinates Analysis (PCoA) with Bray-Curtis distances. The three-dimensional plot model of the principal component analysis (PCA) was done with the scatterplot3d package in R.

### Statistical analysis

Statistical analysis was performed using the Mann-Whitney U test (also known as the Mann-Whitney-Wilcoxon (MWW), Wilcoxon rank-sum test, or Wilcoxon-Mann-Whitney test), which is a nonparametric test of the null hypothesis that it is equally likely that a randomly selected value from one sample will be less than or greater than a randomly selected value from a second sample. The test was used to determine whether two independent samples were selected from populations having the same distribution.

## Results

### Direct visualization of fungal structures in the CNS of PD patients

**Figure 1:**
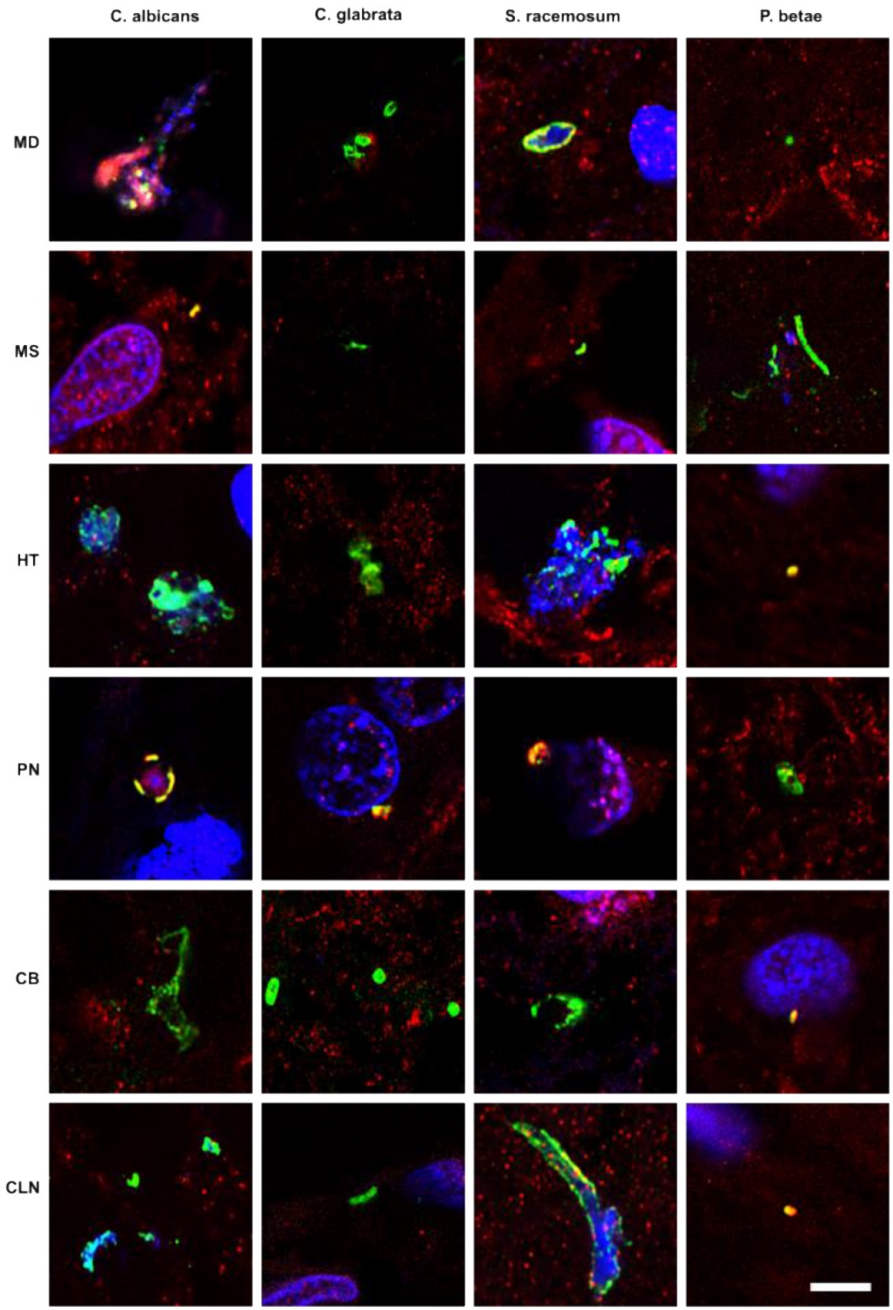
** Fungal structures in different CNS regions from one PD patient (PD1) analyzed by immunohistochemistry.** Six CNS regions (medulla, MD; mesencephalon, MS; hypothalamus, HT; pons, PN; callosal body, CB; and caudate and lenticular nuclei, CLN) of PD1 were processed for immunohistochemistry as described in Materials and Methods. Parafin sections (5 µm) were immunostained with rabbit polyclonal antibodies against *C. albicans, C. glabrata, S. racemosum* and *P. betae* (green). Samples were then immunostained with a mouse monoclonal antibody against human α-tubulin (red). Finally, nuclei were stained with DAPI (blue). Scale bar: 5 µm.

Previous studies from our laboratory employed a battery of rabbit polyclonal antibodies to examine for fungal structures in human brain sections 47-49, 52. As these antibodies are polyclonal, they cross-react with a variety of fungal species, including the one used to raise the antibody. Accordingly, we can obtain direct support for the existence of mycotic structures (yeast-like and hypha) in the CNS, but we cannot make conclusions about the specific fungal species using this technique. As a first attempt to explore the presence of fungi in PD CNS, we examined sections from various brain regions, as described in Materials and Methods. CNS sections were analyzed by double immunofluorescence using one of the following antifungal antibodies: anti-*C. albicans*, anti-*C. glabrata*, anti-*S. racemosum*, and anti-*P. betae* and a second antibody against the human protein α-tubulin. In neurodegenerative diseases and in aged subjects, α-tubulin can be found not only in the cytoplasm, but also in some nuclei of brain cells 59. Notably, a variety of morphologies were revealed in the CNS of patient PD1 by immunohistochemistry and confocal microscopy (Figure 1). Several fungal structures (green) were clearly evident, such as yeast-like cells (see panels MD *S. racemosum* and PN *C. glabrata*), hyphal structures (panels MS *P. betae*, CB *C. glabrata* and CLN *S. racemosum*) and other rounded small bodies (panels MD *C. glabrata*, PN *S. racemosum* and CB *C. glabrata*). DAPI staining (blue) revealed the presence of nuclei in some of these fungal structures. Accordingly, the evidence for fungal infection is based on both the typical mycotic morphologies and their reactivity with anti-fungal antibodies.

To further assess the presence of fungi in these sections, we used two specific rabbit polyclonal antibodies raised against purified fungal enolase and β-tubulin, which do not cross-react with human tissue 56. The anti-enolase antibody (green) was combined with a mouse monoclonal anti-human α-tubulin antibody (red), whereas the anti-fungal β-tubulin antibody (green) was combined with a mouse monoclonal anti-human neurofilament antibody (red). Once again, typical mycotic morphologies could be revealed using these two monospecific antibodies (Figure S1).

**Figure 2:**
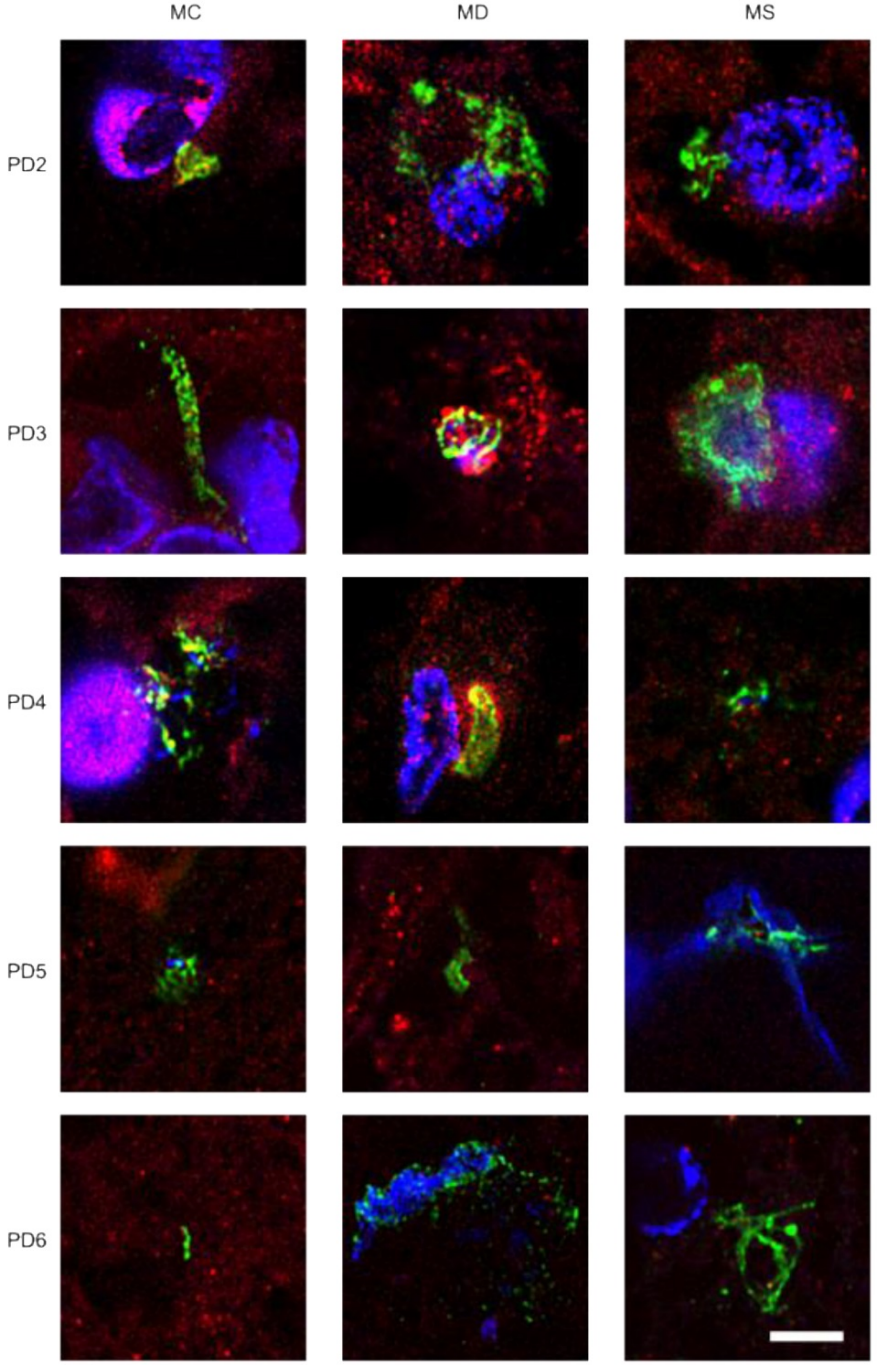
** Immunohistochemistry analysis of CNS regions from five PD patients (PD2-PD6).** Three CNS regions (motor cortex, MC; medulla, MD; and mesencephalon, MS) from five PD patients (PD2-PD6) were inmunostained with a rabbit polyclonal antibody against *C. albicans* (green) and a mouse monoclonal antibody against human α-tubulin (red). Nuclei were stained with DAPI (blue). Scale bar: 5 µm.

To extend this analysis to other patients, three CNS sections (MC, MD and MS) from five PD patients (PD2-PD6) were analyzed by immunohistochemistry and confocal microscopy. Initially, these samples were immunostained with a rabbit polyclonal antibody for *C. albicans* (green) and a mouse monoclonal antibody for human α-tubulin (red). In all cases, there was clear evidence for the presence of fungi in the three CNS regions (Figure 2). Of interest, some forms that immunoreact with the anti-*C. albicans* antibodies appeared to be intracellular (see panels PD3 MS and PD4 MC). We then used an anti-*P. betae* antibody to examine these samples (Figure S2), finding morphologies similar to those observed with patient PD1 with the other antifungal antibodies, which is consistent with the occurrence of fungal infection in the CNS of PD patients. Comparison of these findings with fungi observed in control elderly persons using the same antibodies indicated that the burden of fungal infection was greater in PD as compared with controls 51.

### Intracellular localization of fungi in neural cells

**Figure 3:**
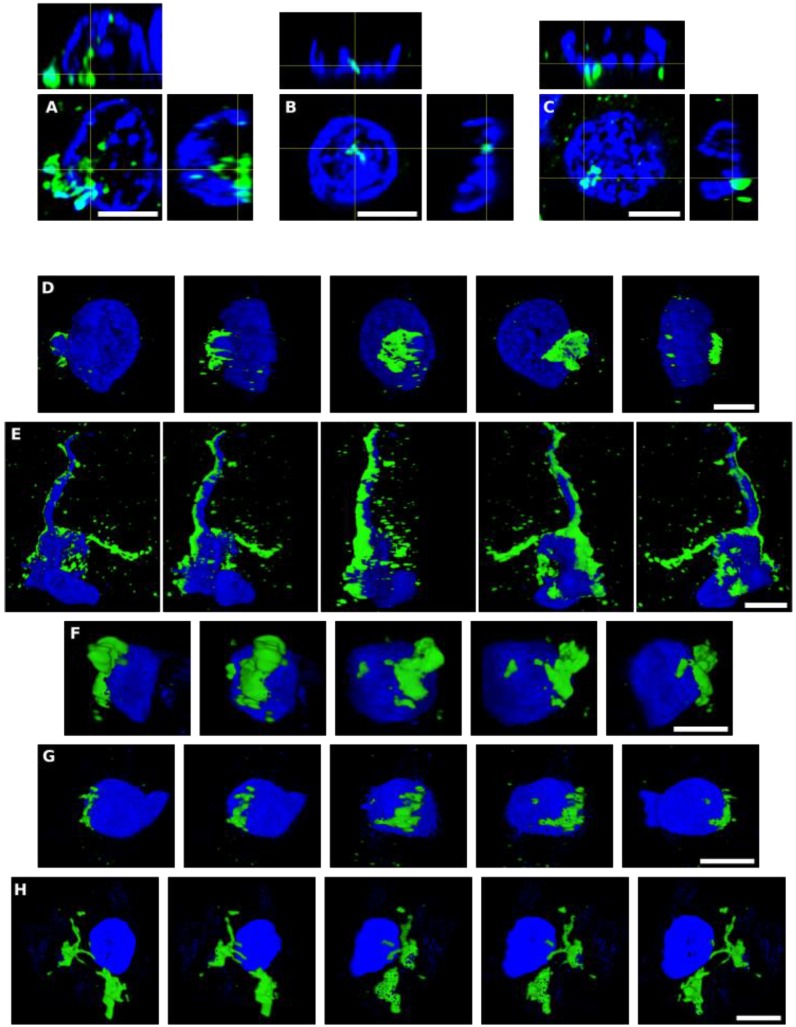
** Orthogonal projections and 3D analyses of CNS sections from PD patients.** Immunohistochemistry was carried out as described in Figure 1. Orthogonal projections (panels A-C) and different stacks from a 3D image (panels D-H) (see also Movies S1-S5) from different sections and PD patients. Panels A and D-F: PD1 and panels B, C and G,H: PD4. Panels A, D-F: CLN region; panels B,C and G: MC region and panel H: MD region. Panels A and D-F: samples were immunostained with an anti-*P. betae* antibody (green) and panels B,C and G,H: samples were immunostained with an anti-*C. albicans* antibody (green). Nuclei were stained with DAPI (blue). All scale bars: 5 µm.

As mentioned, some of the fungal structures observed presented an intracellular location, and in some instances, appeared to be intranuclear. To further examine this possibility, we analyzed orthogonal projections of these specific cells. As shown in Figure 3, some yeast cells could be viewed inside the nucleus or close to it, and hyphal structures could also be detected surrounding the nuclei. Both yeast and hyphae could be stained with DAPI (blue), indicating that they contain nuclei, which was well differentiated from the neural cell nucleus (Figure 3). Finally, video images provided compelling evidence for the intracellular location of the fungal cells (Movie S1, S2, S3, S4 and S5, available online). Of note, the intracellular colonization of human cells by yeast requires that the cell is metabolically active 60-64. Therefore, our observations lend support to the concept that human neural cells were alive during the progression of fungal infection.

### Detection of chitin in PD CNS

**Figure 4:**
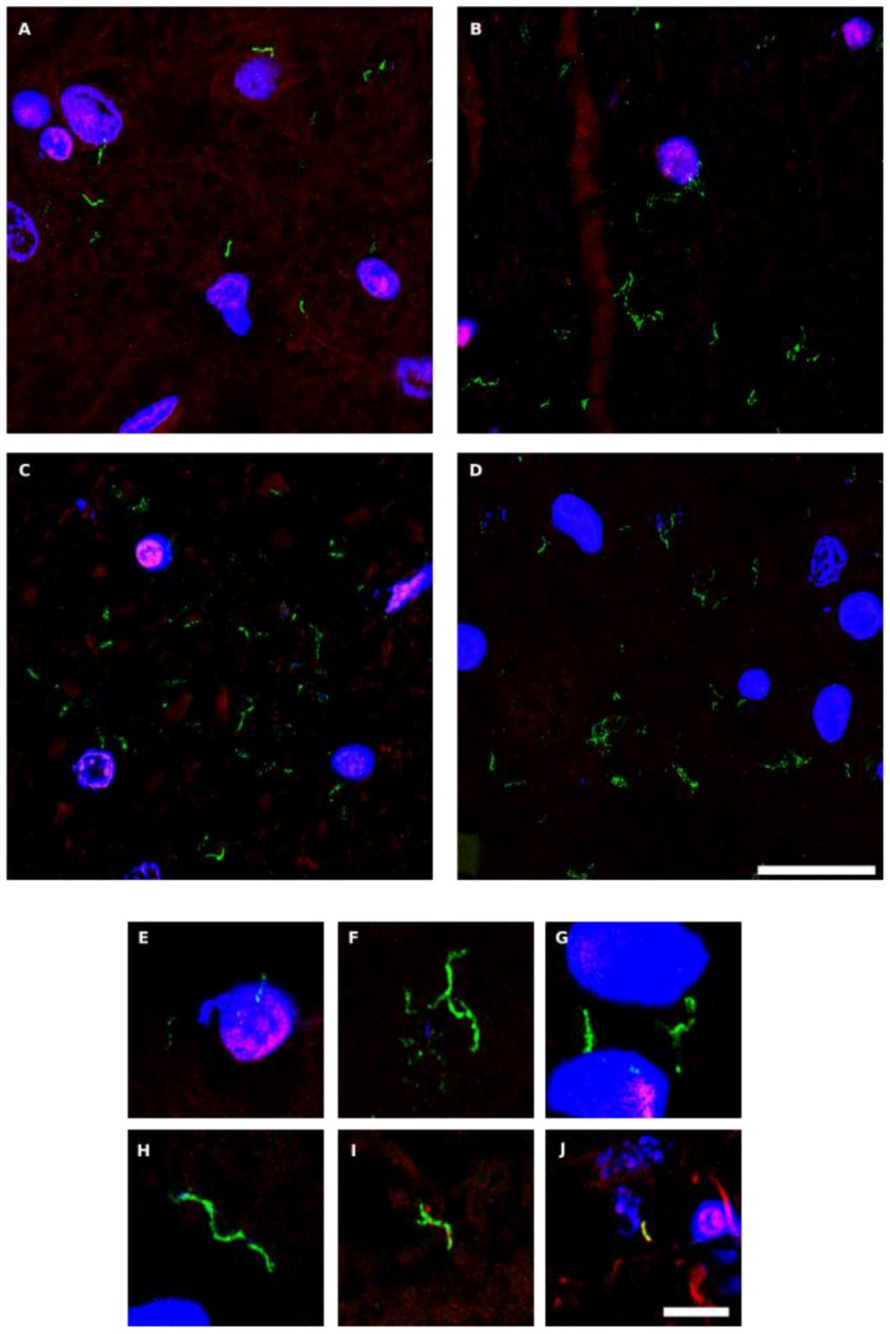
** Hyphal structures in CNS sections of PD patients detected by an anti-chitin antibody.** Several regions from different PD patients were processed for immunohistochemistry analysis as described in Materials and Methods using anti-chitin (green) and anti-human α-tubulin (red) antibodies. Nuclei appear in blue. Scale bar: 20 µm for panels A-D and 5 μm for panels E-J.

The polysaccharide chitin is a typical component of the fungal cell wall and can be detected by means of specific antibodies 65, 66. Because the presence of chitin in human tissues provides persuasive evidence for mycotic cells, we examined for the existence of chitin in CNS sections from six PD patients (PD1-PD6) using a rabbit polyclonal anti-chitin antibody (green), and counterstained with a mouse monoclonal anti-human α-tubulin antibody (red). Strikingly, a number of hyphae could be revealed using the anti-chitin antibody (Figure 4). Consistent with the previous results, some hyphae seemed to be intracellular, whereas others were detected in intercellular spaces and, in some instances, DAPI staining (blue) of the nuclei of these hyphae was evident. In addition to these typical mycotic structures, other morphologies and diffuse material were observed with the anti-chitin antibody (Figure S3). Importantly, some rounded fungal cells were found both intranuclearly and outside of the nucleus (Figure S3, panels PD1 MS, PD3 MC and MS). In conclusion, the evidence for cells containing chitin in the CNS of PD patients supports the existence of fungal infection. The various sizes and morphologies observed likely reflect the existence of different fungal species.

As a control for this analysis we used CNS sections from four elderly controls. As shown in Figure S4, there was no sign of chitin immunopositivity in these sections, demonstrating that fungal colonization is not detectable in these subjects with the anti-chitin antibody.

### Identification of fungal species by nested PCR

The precise identification of the fungal species present in neural tissue can be achieved by DNA sequencing. To this end, total DNA was extracted from frozen CNS tissue samples and nested PCR was used to amplify specific fungal DNA sequences. This sensitive technique was necessary because the bulk of DNA obtained is human in origin and the amount of microbial DNA should be very low. A robust method to examine for different fungal species potentially present in these samples is amplification of the intergenic sequences (ITS1 and ITS2) that exist between the rRNA genes (see scheme Figure S5, panel A). We have previously established primer pairs that amplify fungal ITS1 and ITS2 49, 53. In our experience, some fungal species are preferentially amplified by this technique depending on the primers employed, and therefore the use of different primer pairs can be complementary to detect different species.

Using extracted DNA from several regions of the six PD patients (PD1-PD6), nested PCR was performed to amplify both the ITS1 and the ITS2 regions (Figure S5, panel B). Amplified DNA fragments were separated in agarose gels, and the corresponding bands were extracted and sequenced to identify the fungal species. Importantly, no DNA fragments were amplified in controls of the PCR assay, with no DNA, and in a control for DNA extraction. Also, in some instances no amplification was observed in some of the samples tested, suggesting that the amount of fungal DNA in these samples was below the threshold level for the reaction. Sequencing of the different DNA fragments revealed a variety of fungal species (Table 1). Some species were found only after amplification of ITS1, whereas others were identified with primers for ITS2. Also, several genera including *Cladosporium*, *Malassezia* or *Penicillium* were occasionally detected with both PCR assays. Nevertheless, the detection of a given species by this assay demonstrates its presence in a given sample. The conclusion from these data is that a variety of fungal species can be detected in each PD patient. These species can differ depending on the CNS region examined and also they can vary from patient to patient.

### Next-generation sequencing of fungal DNA extracted from PD CNS

**Figure 5:**
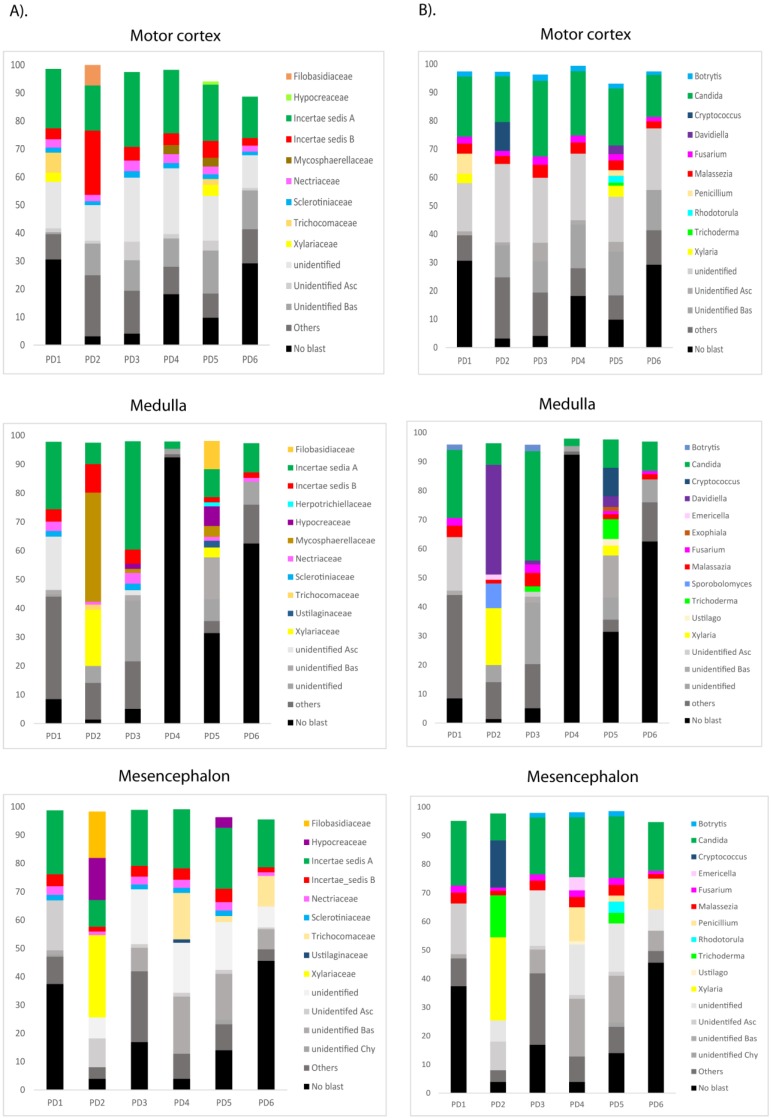
** Distribution of fungal families and genera in six PD patients after NGS analyses.** Computational analyses of the sequences obtained on the Illumina platform using Qiime classified the data fungal families and genera. Panel A shows the results of fungal families obtained from motor cortex, medulla and mesencephalon of PD patients. Panel B shows the results of fungal genera obtained from motor cortex, medulla and mesencephalon of PD patients. Asc: Ascomycota, Bas: Basidiomycota, Chy: Chytridiomycota.

The most sensitive technique to gain further insight into the mycobiome in the CNS of PD patients is NGS. Accordingly, we used the Illumina platform to sequence DNA samples using the primers described in Materials and Methods. The number of sequences carried out in each sample varied from 614,956 to 905,022. The fungal species detected by this sensitive technique are listed in Table S2. Only the species above 1% were included. The distribution of fungal families and species in the six patients, considering the three CNS regions MC, MS and MD, are shown in Figure 5. Importantly, several genera such as *Botrytis*, *Candida, Fusarium and Malassezia* were found in several patients and, in some instances, their percentages were rather high, in some instances these percentages were about 10-30% (Figure 5, panel B) Some of these genera are common to AD, whereas other genera such as *Fusarium, Rhodotorula* and *Trichoderma* have been detected in PD but not in AD 51, 53. For instance, the genus *Alternaria* was prominent in some AD patients, but it was not found in PD.

**Figure 6:**
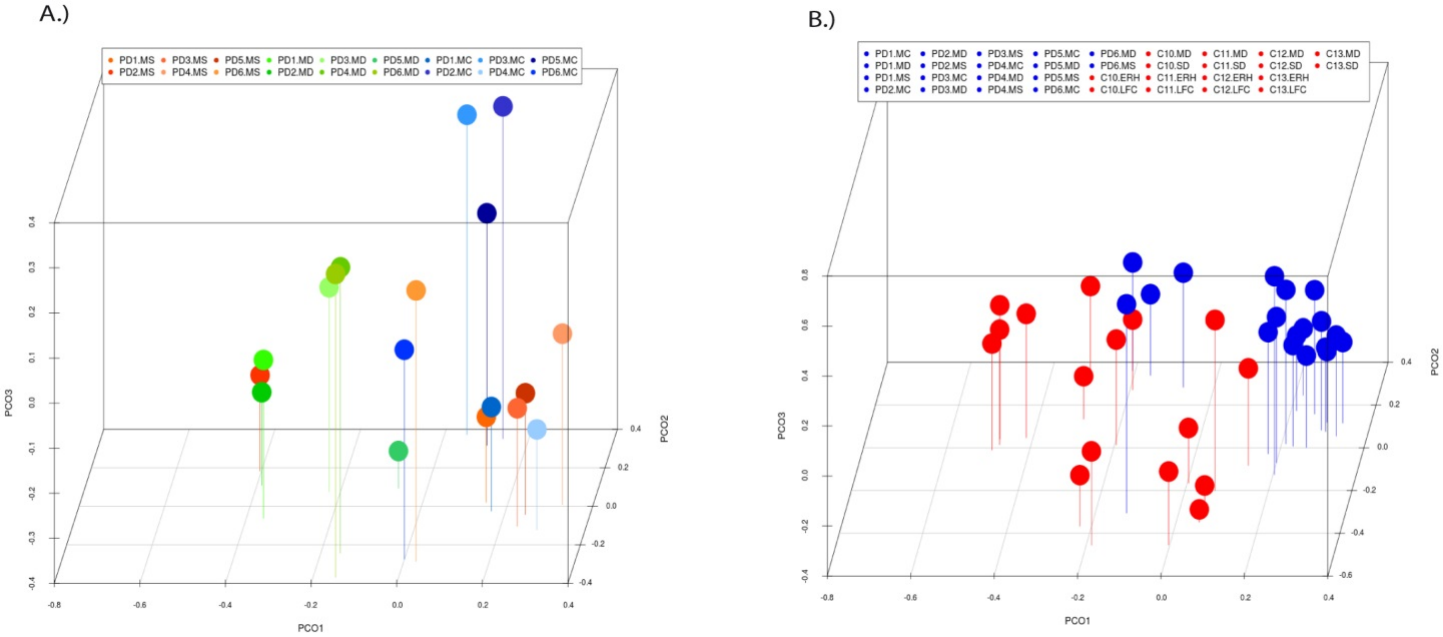
** Principal component analysis of the fungal species detected in different CNS regions of PD patients.** 3D principal component analysis scatter plots of PD patients and control subjects. Panel A shows the distribution between different regions of six PD patients. Panel B shows the distribution between PD patients (plots in blue) and control subjects (plots in red). The UniFrac method was used to calculate this parameter. MC, motor cortex; MD, medulla; MS, mesencephalon; LFC, lateral frontal cortex; ERH, enthorinal cortex; and SC, spinal cord.

PCA of the data provided a number of interesting results. For example, when only the different CNS regions from PD patients were analyzed, the fungi appeared to be preferentially segregated in distinct regions (Figure 6, panel A). This finding suggests that the mycobiota in each region can be differentiated by this analysis. Furthermore, statistical analysis showed significant differences between the three regions (MC, MD and MS): the p-value between MC and MD was 0.000034, the p-value between MC and MS was 0.00000006, and the p-value between MD and MS was 0.0032. In addition, when the mycobiome from PD and control subjects were compared, the results obtained were also very significant, since the fungi present in PD and controls appeared clearly separated (Figure 6, panel B). These observations further support the concept that the mycobiota from different CNS regions and controls is not due to post-mortem contamination. Most likely, these fungi are present before death because they are different in several CNS regions from PD and controls.

### Bacterial peptidoglycan in brain tissue from PD patients

**Figure 7:**
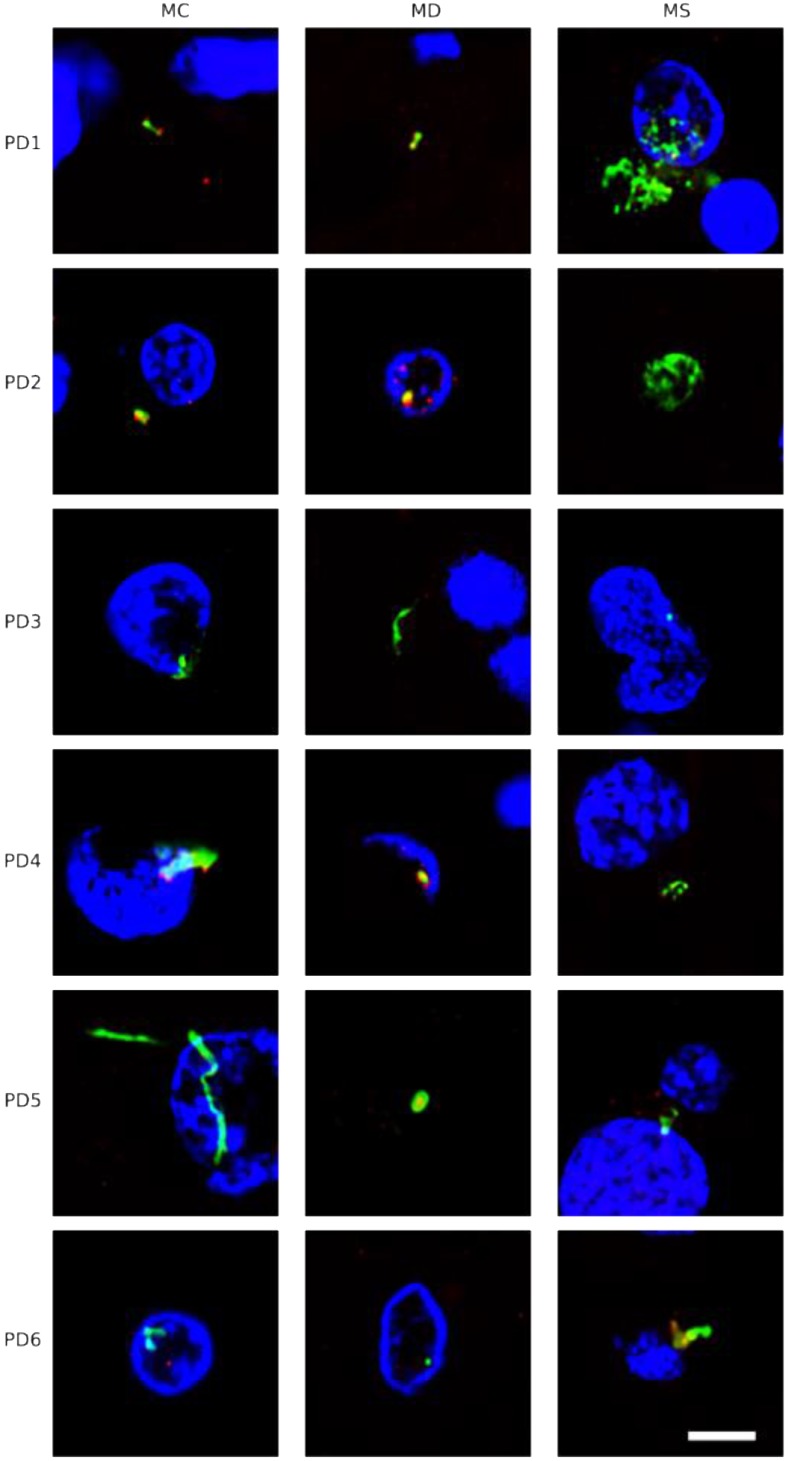
** Detection of peptidoglycan in CNS sections from PD patients using immunohistochemistry.** PD samples were processed as described in Materials and Methods. Samples were first immunostained with an anti-peptidoglycan antibody (green) and then with an anti-*C. albicans* antibody (red). DAPI staining of nuclei appears in blue. Scale bar: 5 µm.

To our knowledge, there are no data showing direct visualization of bacteria in the CNS of PD patients, despite the suggestion by several groups that some bacteria could be involved in the pathology of this disease 24, 25, 30, 32. Moreover, it is known that lipopolysaccharide injection can induce symptoms similar to Parkinsonism 67, 68. Given this, we considered it interesting to examine for the existence of bacteria in the CNS of PD patients using immunohistochemistry. We first employed a mouse monoclonal antibody against peptidoglycan, which is an abundant component of the cell wall of Gram-positive bacteria. We used this in combination with the rabbit polyclonal antibody against *C. albicans*. A number of prokaryotic cells were apparent in the CNS sections of the six PD patients tested (Figure 7). Curiously, also some mycelial morphologies stained positive with the anti-peptidoglycan antibody (Figure 7, panel PD5 MC, shown in green). Indeed, this type of morphology is found in a number of bacterial species including some actinomycetes. Notably, some prokaryotic cells were also found intracellularly and some of these cells seemed to display an intranuclear location (Figure 7, panels PD1 MS, PD2 MD, PD3 MC and PD6 MC). We then used a second anti-bacterial antibody, a rabbit polyclonal antibody against *Chlamydophila pneumoniae* (green), that recognizes the major outer porin (Figure S6), but also cross-react with other bacteria 47. This antibody was combined with a rat polyclonal antibody against *Trichoderma viride* (red). Consistent with the above findings, a number of punctate bodies were revealed using this bacterial antibody. Thus, we conclude that a variety of prokaryotic structures can be observed in the CNS of PD patients. These findings suggest that polymicrobial infections likely exist in the neural tissue of PD.

### Identification of bacterial species in the CNS of PD patients

The above findings pointed to the possibility that different bacteria are present in the CNS of PD patients. To confirm this, we used nested PCR analyses as before with primer pairs to amplify the IGS region between the rRNA genes (see scheme Figure S7, panel A). As expected, a variety of DNA fragments was found after separation in agarose gels (Figure S7, panel B). Each DNA fragment was extracted and sequenced, revealing a number of bacterial species (Table 2). Some of these bacterial species have also been previously found in our studies of the microbiota in the CNS of AD patients 47, for example the finding of *Cutibacterium acnes*.

### NGS of bacterial DNA extracted from PD CNS

**Figure 8:**
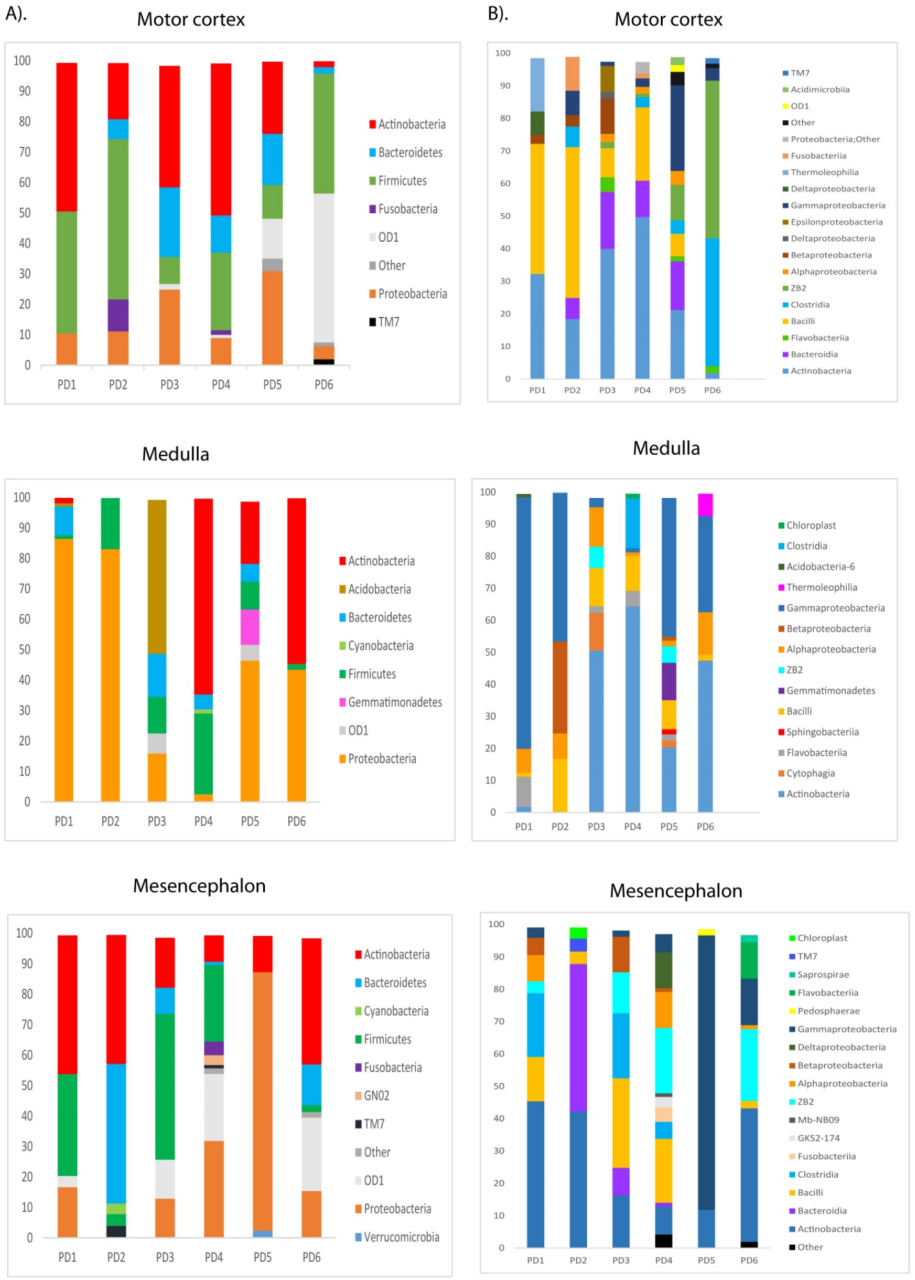
** Distribution of bacterial phyla and classes in CNS samples from PD patients.** Computational analyses of the sequences obtained using NGS were carried out with Qiime program. Bacterial phyla and classes obtained with this program are shown. Panel A shows bacterial phyla detected in MC, MD and MS of PD patients. Panel B shows bacterial classes detected from MC, MD and MS of PD patients.

To gain further insight into the microbiome in the CNS of PD patients, we used NGS as before. Previous studies from several laboratories, including ours, have described a great variety of bacterial species in the CNS of patients with neurodegenerative diseases 47, 51, 69-71. Consistent with these findings, our present study also detected a broad range of bacteria in the different CNS regions of PD patients (Table S3). Notably, significant differences were found between the bacteria in PD and those previously identified in AD or ALS patients 50, 51, and some bacteria seem to be more representative of PD patients. For instance, the genera *Streptococcus* mainly appeared in the MC, and *Pseudomonas* was identified in the MD of some patients, whereas *Acinetobacter* was found in all three CNS regions of PD5 (MC, MD and MS). Curiously, the family *Methylobacteriaceae* was not identified in PD, although it was prominent in patients with AD or ALS (Alonso et al., 2017a, Alonso et al., 2018b). The phyla and classes of bacteria from the three CNS regions from the six PD patients are shown in Figure 8. Of interest, the phylum *Actinobacteria* was mainly found in MC and MS, whereas *Proteobacteria* was more abundant in MD.

**Figure 9:**
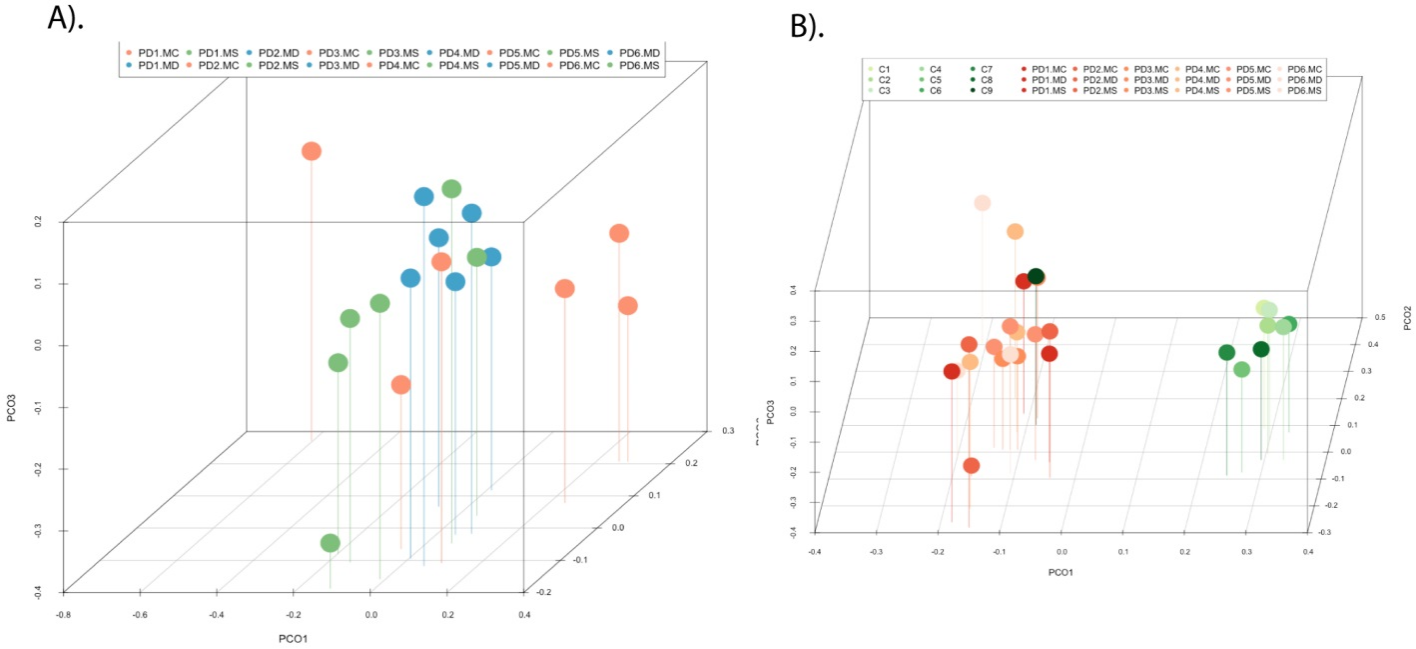
** Principal component analysis of bacterial species in different CNS regions from six PD patients.** 3D principal component analysis scatter plots of PD patients and nine control subjects. Panel A shows the distribution between different regions of six PD patients. Panel B shows the distribution between PD patients (plots in orange) and control subjects (plots in green). The UniFrac method was used to calculate this parameter. MC, motor cortex; MD, medulla; MS, mesencephalon; ERH, enthorinal cortex.

PCA indicated that the segregation of bacteria between the three CNS regions from PD was significantly different in MD and MS (Figure 9, panel A). Furthermore, when the comparison was done between PD patients and nine control subjects 51, the findings were also very significant. Indeed, PCA of bacteria present in PD and controls showed that they were clearly separated, with the exception of C9 (Figure 9, panel B). These observations clearly indicate that the bacteria that form part of the CNS microbiome can be differentiated from control subjects.

### Evidence for fungal and bacterial antigens in *corpora amylacea*

**Figure 10:**
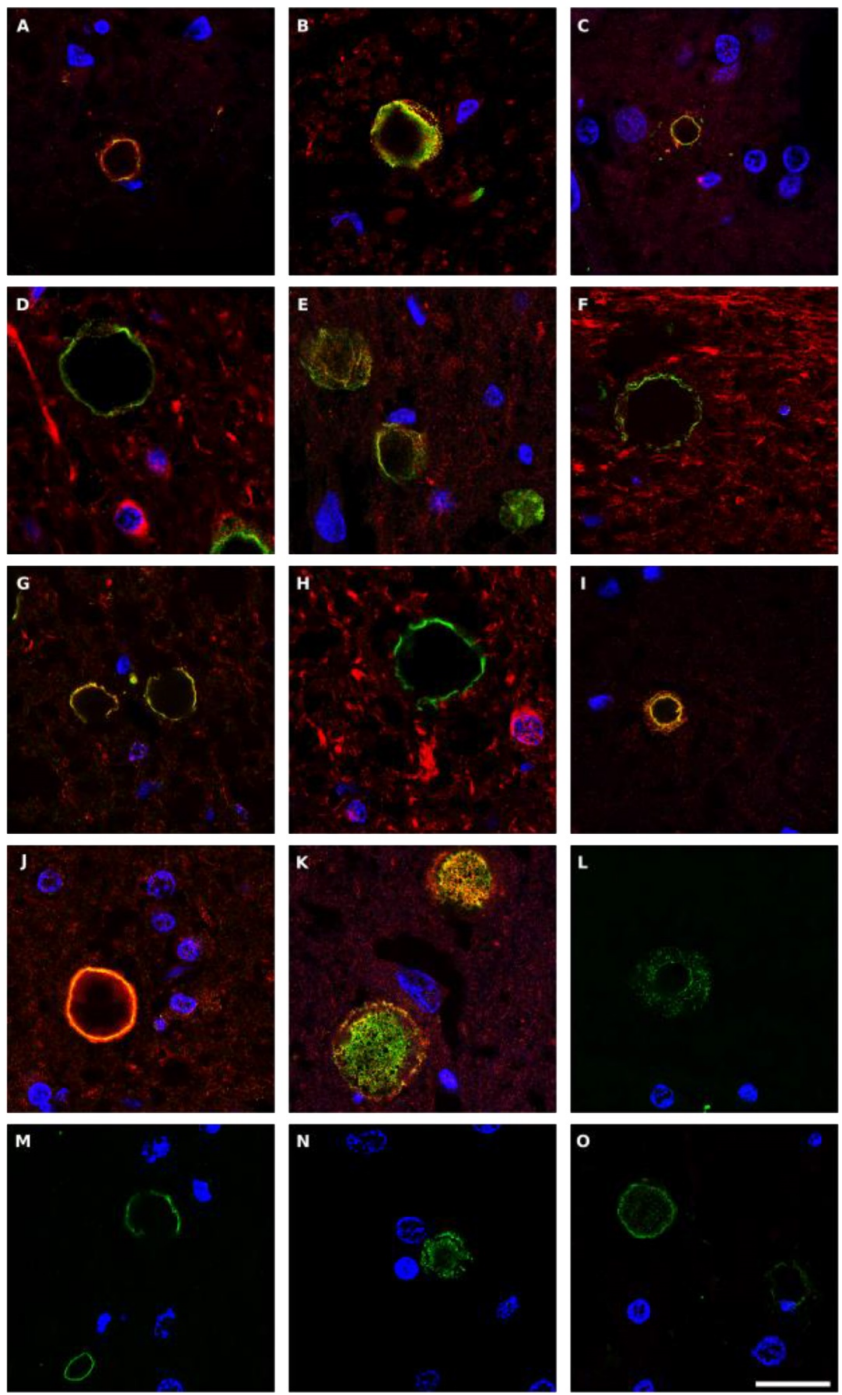
** Fungal antigens in *corpora amylacea* detected in CNS sections from PD patients.** Different sections from PD patients were incubated with rabbit polyclonal antibodies against *C. albicans* (panels A-D), *P. betae* (panels E-H),* S. racemosum* (panels I-K) and chitin (L-O), shown in green, and a monoclonal antibody against human α-tubulin, shown in red. Panels A, E-G and I-K: patient PD1; panels B and L: PD2; panels C and M: PD3; panels N and O: PD4 and panels D and H: PD5. Panels A, C, L and N: motor cortex; panels B, F and J: mesencephalon; panels D and E, H-I, M and O: medulla; panel G: pons; and panel K: hypothalamus. DAPI appears in blue. Scale bar: 20 µm.

Corpora amylacea (CA) are glycoproteinaceous rounded bodies of 10-50 μm in diameter that appear in aged subjects, and are much more abundant in some neurological diseases including PD 55. CA mostly contain polyglucans (about 85%) with a minor proteic component (4%) and different calcium salts, principally calcium phosphate and calcium oxalate 72, 73. We recently established that purified CA from AD brains contain fungal and bacterial proteins, as detected by proteomic analysis 74. In addition, fungal antigens could be detected in CA of CNS samples from several neurodegenerative diseases 55. Given this, we next questioned whether fungal and bacterial antigens were present in CA from PD patients. Double immunofluorescence staining was performed with an anti-fungal antibody (anti-*C. albicans*, anti-* P. betae*, anti-* S. racemosum* or anti-chitin) (green) and a monoclonal antibody against human α-tubulin (red). Results showed that antigens were clearly trapped in CA and they were mostly located in the envelope of these structures (Figure 10). Some punctate fungal material was also observed in the inner part of CA. These observations are consistent with the concept that during the formation of CA, fungal and cellular proteins that are secreted to the medium are trapped. Also, production of cell debris from microbial destruction or neural death can be recruited by CA.

We repeated this analysis for bacterial peptidoglycan (Figure S8). In this case, the mouse monoclonal antibody against peptidoglycan (green) was combined with the rat polyclonal antibody against *T. viride* (red). The presence of peptidoglycan in CA was evident, and was mostly found in the envelope of CA. Interestingly, some CA were immunostained with the anti-*T. viride* antibody, revealing that CA are heterogeneous bodies in which the protein composition of each CA can vary 55. This may be due to the infection of each PD patient by different microbes or to specific locations in the CNS of each particular microbial species due to their tropism. Collectively, these findings demonstrate that CA from PD contain fungal and bacterial antigens. Moreover, since CA are established over long periods of time, most probably years, the fungal and bacterial antigens were likely trapped during CA formation. Therefore, these mixed fungal and bacterial infections existed in the CNS of PD patients some time before their death.

## Discussion

One of the most salient features of PD pathology is neuroinflammation 20, 22. Indeed, microglia and astrocyte activation, in addition to lymphocyte infiltration and cytokine production (IL-1β, IL-2, IL-4, IL-6, IL-10, TNF-*α*, and IFN-*γ*), have been documented in PD 20, 75-77 Some researchers interpret the inflammation as a result of neural death and the appearance of cell debris that should be cleared from the brain in PD patients. However, an alternative and plausible possibility for neuroinflammation is the existence of chronic and progressive polymicrobial infection, as illustrated by our present findings. This would also explain the signs of systemic inflammation that appear even years before motor symptoms. Thus, it is possible that these polymicrobial infections colonize different tissues and organs of the human body including the CNS, eliciting an immune response that could be reflected in the blood serum. For instance, elevated levels of C reactive protein have been associated with the risk of death and predicted prognosis of patients with PD 78, 79.

The concept that fungal or bacterial infections might trigger inflammation in PD has been advanced by several groups 24, 25, 27, 30, 32, although evidence to support these infections was not provided. In the present study work, we report the first direct evidence of fungi and bacteria in PD neural tissue, as demonstrated by immunohistochemistry using specific antibodies, which also revealed that some of these microbial cells are inside neurons, supporting the concept that patients were alive during the infection. Moreover, the comprehensive identification of fungal and bacterial species by NGS provides a detailed picture of the heterogeneity of these polymicrobial infections in each PD patient. This compelling evidence is consistent with the idea that fungal and bacterial infections take place during disease progression. We believe that these findings will open new horizons on the role of these polymicrobial infections in the inflammation and clinical symptoms observed in the CNS of PD patients. The heterogeneity of the symptoms found in each patient might be a consequence of the variety of microbes affecting the CNS. Moreover, it could be possible that these infections may target other organs or tissues in some patients, leading to systemic inflammation. Indeed, a variety of clinical symptoms and pathologies beyond the CNS, such as dermatitis, have been associated with PD 80. In conclusion, the great variety of fungi and bacteria, their combinations, and the potential production of toxic substances may constitute the basis to understand the various pathological features of PD patients.

As regards to the pathway followed by polymicrobial invasion, several possibilities can be envisaged, which are not mutually exclusive. It should be possible that the initial infection by a microbial species facilitates further colonization by other oportunistic microbes. These fungi and bacteria could spread to different brain regions depending on their tropism. Therefore, a complex picture arises in which each individual patient with the corresponding microbiota is progressively infected by a variety of microbial species. The most external part of the CNS is represented by bipolar sensory neurons that connect the olfactory mucosa with the brain. Thus, the dendrites of these neurons are in the mucosa and project the axon into the olfactory bulb. One of the earliest symptoms in the vast majority of PD patients is the loss of smell, which is followed by a degeneration of the olfactory bulb 10, 81. Since this mucosa is exposed to environmental microorganisms, it is possible that their portal of entry into the CNS is *via* the olfactory neurons. Another possibility for the entry of microbes is by infection of the enteric nervous system, which could propagate to the CNS through the vagus nerve 82. In support of this idea, PD patients present with gastrointestinal inflammation and other anomalies, such as constipation, which usually precedes (for many years) the motor symptoms typical of PD 83, 84. Also, the fact that the gut microbiota can influence microglia activation and some PD symptoms agrees with the concept that the intestinal mucosa may be the site of entry of the different microbes, identified in this work.

Similarities have been observed between PD and other neurological diseases, particularly AD 42-44. For instance, cognitive decline, sleep alterations, depression and dementia, among others are shared by a significant percentage of AD and PD patients. Moreover, amyloid deposits and intracellular phosphorylated tau and α-synuclein have been described both in AD and PD 45, 85, 86. Increasing evidence points to the idea that AD can be caused by microbial infections 47, 51, 87 and we have provided detailed identification of fungi and bacteria in AD brains 51, 53, 55-57. This burden of microbial infections in AD was higher as compared to control individuals 51. Interestingly, our present findings lend support to the concept that AD and PD patients also share polymicrobial infections in the CNS. These infections may affect different brain regions and, therefore, can account for the differences in motor symptoms found in PD. Other differences between AD and PD are the specific microbial infections that exist in each patient, which could reflect differences in their microbiota. For instance, significant percentages of the genus *Alternaria* was detected in AD, but not in PD, whereas the genera *Fusarium*, *Xylaria* and *Trichoderma* were more prominent in PD, as compared with AD. Also, high levels of the bacteria *Streptococcus* and *Pseudomonas* have been detected in PD, but not in AD or ALS patients. Curiously, the genus *Burkholderia* identified in AD was not found in PD. On the other hand, the family *Methylobacteriaceae* was very prominent in AD and ALS patients, but not in PD patients. Clearly, the genetic background of each patient can contribute to the susceptibility and tropism of fungal and bacterial species, leading to differences in the neuropathology.

The consequences of our observations are very promising for the future treatment of these neurodegenerative illnesses. Since there are a number of potential biomarkers and prodromal symptoms, these patients could be treated before motor problems or dementia are evident. In this regard, these diseases could be treated/prevented by administration of safe antifungal and antibacterial compounds years before the alterations or destruction of brain regions, which would make it more difficult to reverse the clinical symptoms. Therefore, with our present knowledge it should be possible to predict the appearance of these diseases with time and to combat them with efficient therapies that have already been approved for clinical use.

## Supplementary Material

Supplementary figures and tables.

Supplementary movie 1.

Supplementary movie 2.

Supplementary movie 3.

Supplementary movie 4.

Supplementary movie 5.

## Figures and Tables

**Table 1:** Fungal species detected by nested PCR

REGION ITS1			REGION ITS2	
Species	Patients		Species	Patients
*Alternaria alternata*	PD2(MC)		*Aspergillus vitricola*	PD1(PN)
*Cladosporium sp*	PD1(MS);PD2(MD,MS);PD4(MC);PD5(MC)		*Aureobasidium pullulans*	PD5(MD)
*Cryptococcus curvatus*	PD1(MD,PN,MS)		*Candida albicans*	PD3(MS)
*Fusicolla aqueductum*	PD4(MS);PD6(MD)		*Candida sp*	PD5(MS)
*Malassezia globosa*	PD6(MC)		*Cladosporium cladosporoides*	PD1(CB);PD2(MD);PD3(MD);PD4(MC);PD6(MC)
*Microdochium nivale*	PD1(MD)		*Cladosporium puyae*	PD2(MS)
*Penicillium chrysogenum*	PD3(MC); PD4(MD)		*Malassezia globosa*	PD1(MD);PD5(MD,MS);PD6(MD)
*Penicillium digitatum*	PD6(MS)		*Malassezia restricta*	PD2(MC,MS); PD4(MS)PD6(MS)
*Phoma sp*	PD3(MS)		*Penicillium chrysogenum*	PD5(MC)
*Trichosporon mucoides*	PD5(MD)		*Penicillium sp*	PD1(MS)
*Uncultured fungus*	PD1(HT,CLN)		*Uncultured malassezia*	PD1(PN,MS,HT);PD2(MD)

MC: Motor cortex; MD: Medulla; MS: Mesencephalon; HT: Hipothalamus; PN: Pons; CB: Callosal body; CLN: Candate and lenticular nuclei

**Table 2:** Bacterial species detected by nested PCR

Species	Patients
*Cutibacterium acnes*	PD1(MD,MS,PN,HT,CLN,CB)PD3(MC);PD5(MC, MD,MS); PD6 (MC,MD)
*Niastella Koreensis*	PD4 (MS)
*Rhotia dentocariosa*	PD4(MC)
*Sthapylococcus espidermidis*	PD1( CLN)
*Streptococcus oralis*	PD1(MC)
*Streptococcus pneumonie*	PD1(HT)
