# Supplementary material for: Parkinson's Disease: A Comprehensive Analysis of Fungi and Bacteria in Brain Tissue
Source: Int J Biol Sci. 2020 Feb 10;16(7):1135–52. doi: 10.7150/ijbs.42257 (PMC7053320; doi:10.7150/ijbs.42257)
Supplement: Supplementary file 1 — Supplementary figures and tables. [file ijbsv16p1135s1.pdf]

PARKINSON'S DISEASE: A COMPREHENSIVE ANALYSIS OF FUNGI AND BACTERIA IN BRAIN TISSUE

Diana Pisa, Ruth Alonso and Luis Carrasco[*]

Centro de Biología Molecular "Severo Ochoa" (CSIC-UAM). c/Nicolás Cabrera, 1. Universidad Autónoma de Madrid. Cantoblanco. 28049 Madrid. Spain.

[+]DP and RA contributed equally to this work.

[*]Corresponding author

Email address: lcarrasco@cbm.csic.es. Telephone number: +34 91 497 84 50

Running title: Parkinson's disease and microbial infection

Key words: Parkinson's disease; neurodegenerative diseases; polymicrobial infections; fungal infection; next-generation sequencing

Supplementary Figure 1. Fungal structures of CNS sections from PD1 revealed with anti-fungal enolase and anti-fungal tubulin antibodies. Immunohistochemistry analysis of CNS sections from patient PD1 was performed by confocal microscopy as detailed in Materials and Methods. Sections were immunostained with rabbit polyclonal antibodies against fungal β-tubulin and enolase (green) and human neurofilaments or α-tubulin (red). Panels A–E: anti-fungal β-tubulin and anti-human neurofilaments antibodies. Panels F–R: anti-fungal enolase and anti-human α-tubulin antibodies. Panels A–D and R: caudate and lenticular nuclei. Panels E and P–Q: callosal body. Panels F and O: pons. Panels G–J: medulla. Panels K and L: mesencephalon. Panels M and N: hypothalamus. DAPI staining appears in blue. Scale bar: 5 μm.

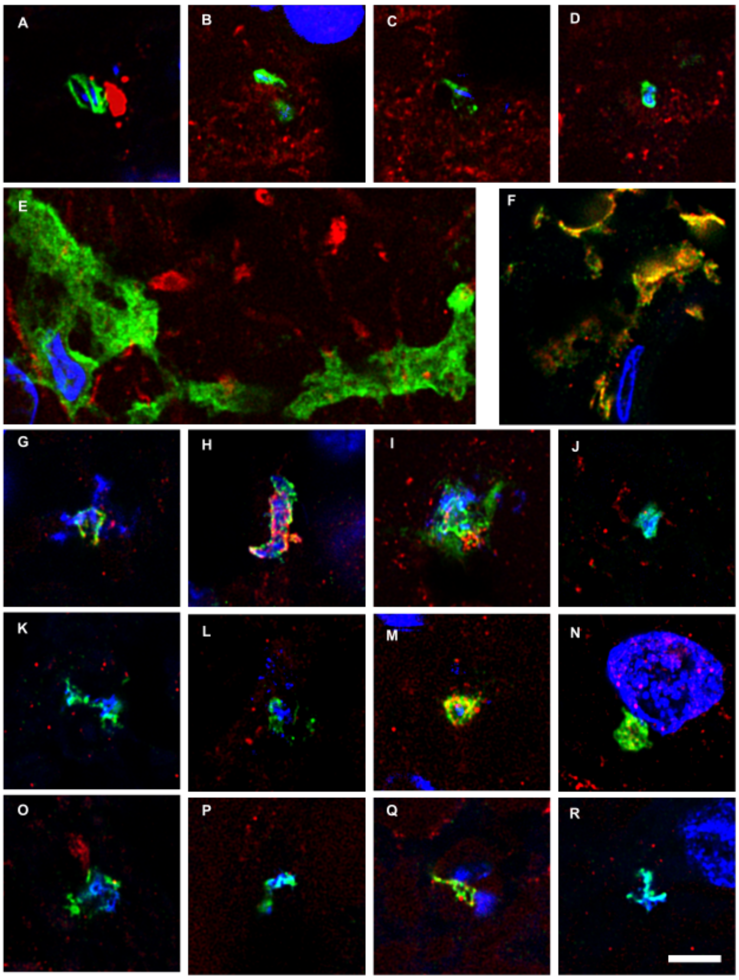

Supplementary Figure 2. Immunohistochemistry with an anti-*P. betae* antibody of CNS sections from five PD patients. Three CNS regions (motor cortex, MC; medulla, MD; and mesencephalon, MS) from five PD patients (PD2–PD6) were immunostained with a rabbit polyclonal antibody against *P. betae* (green) and a mouse monoclonal antibody against human α–tubulin (red). Nuclei were stained with DAPI (blue). Scale bar: 5 μm.

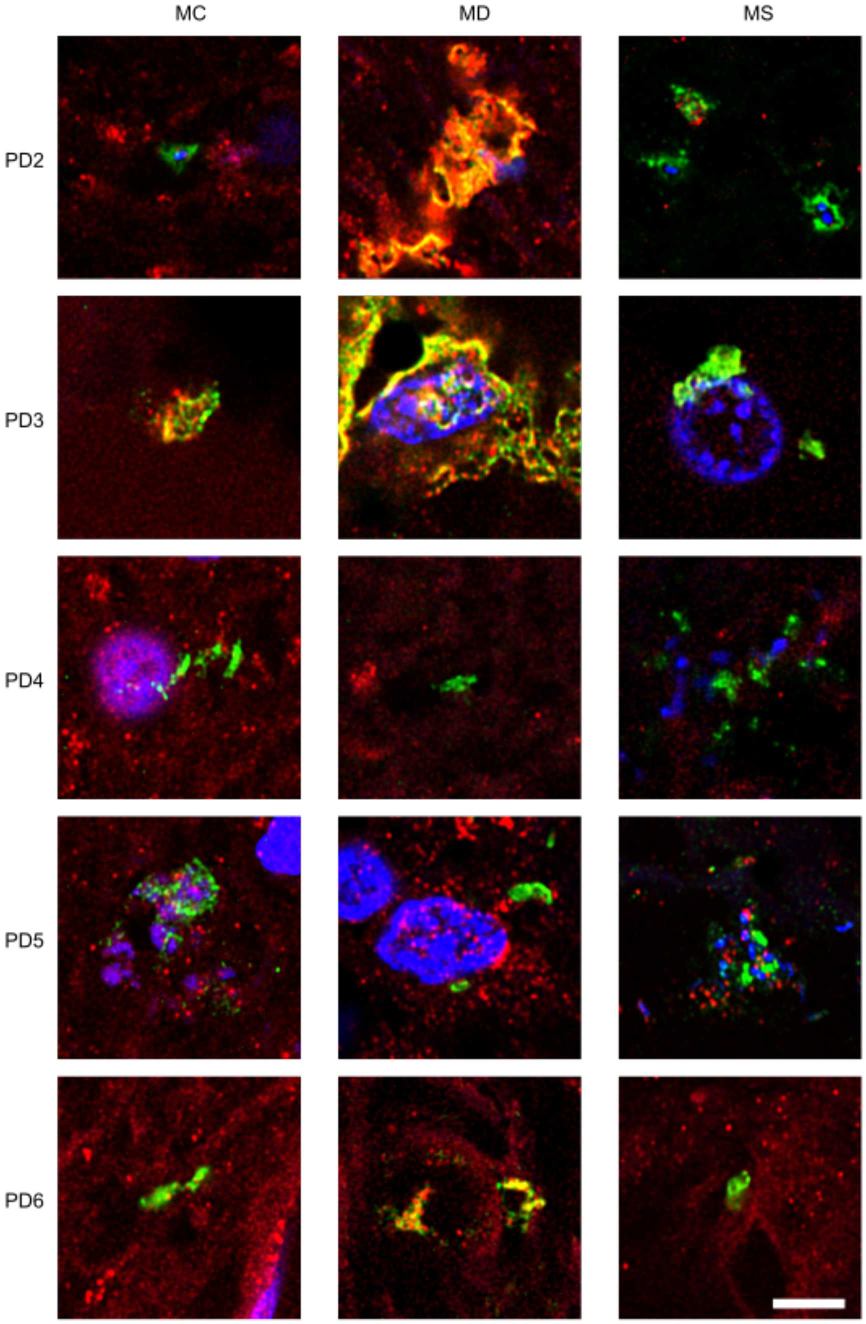

Supplementary Figure 3. Immunohistochemistry of CNS sections from six PD patients using an anti-chitin antibody. Different regions of six PD patients were incubated using anti-chitin (green) and anti-human α-tubulin (red) antibodies. Nuclei appear in blue. Scale bar: 5 μm.

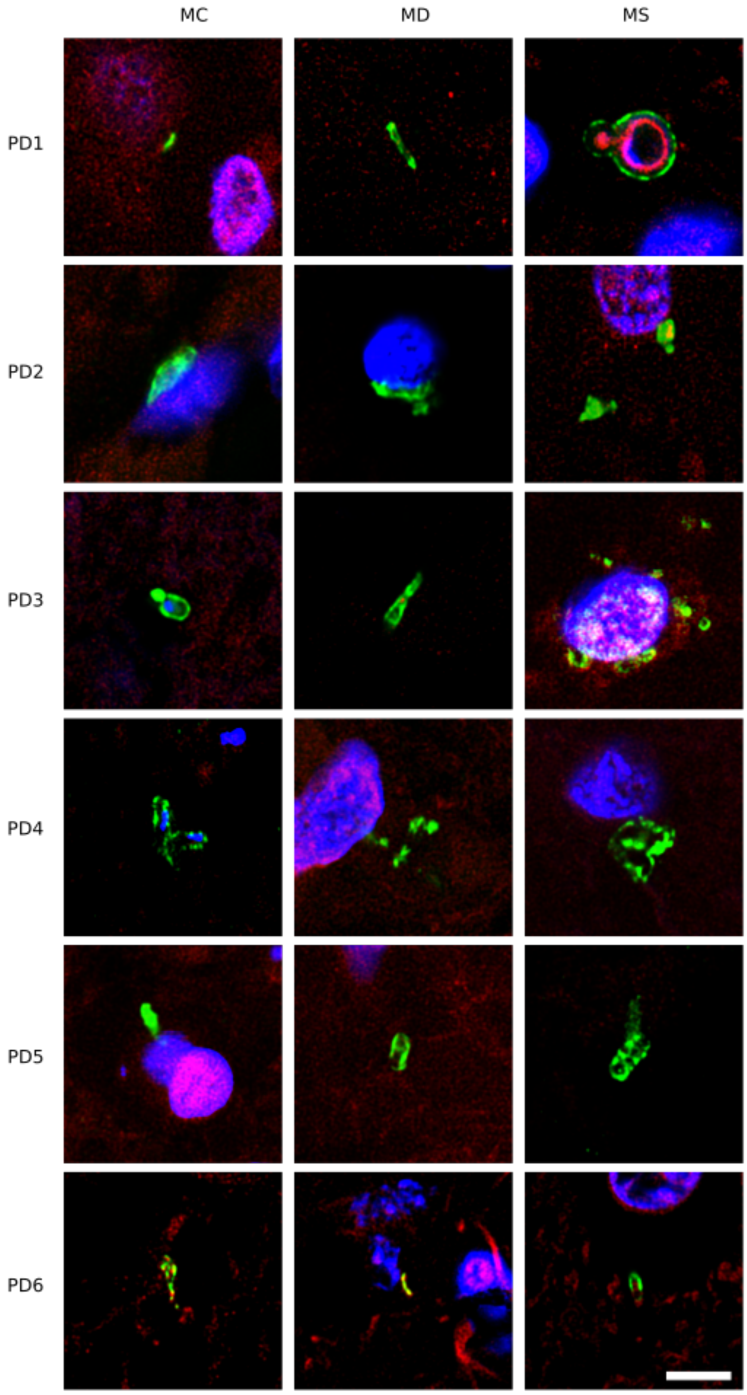

Supplementary Figure 4. Immunohistochemistry of CNS samples from control subjects using an anti-chitin antibody. Different regions of four control subjects were incubated using anti-chitin (green) and anti-human α-tubulin (red) antibodies. Nuclei appear in blue. C1 and C2: spinal cord. C3 and C4: medulla. Scale bar: 20 μm.

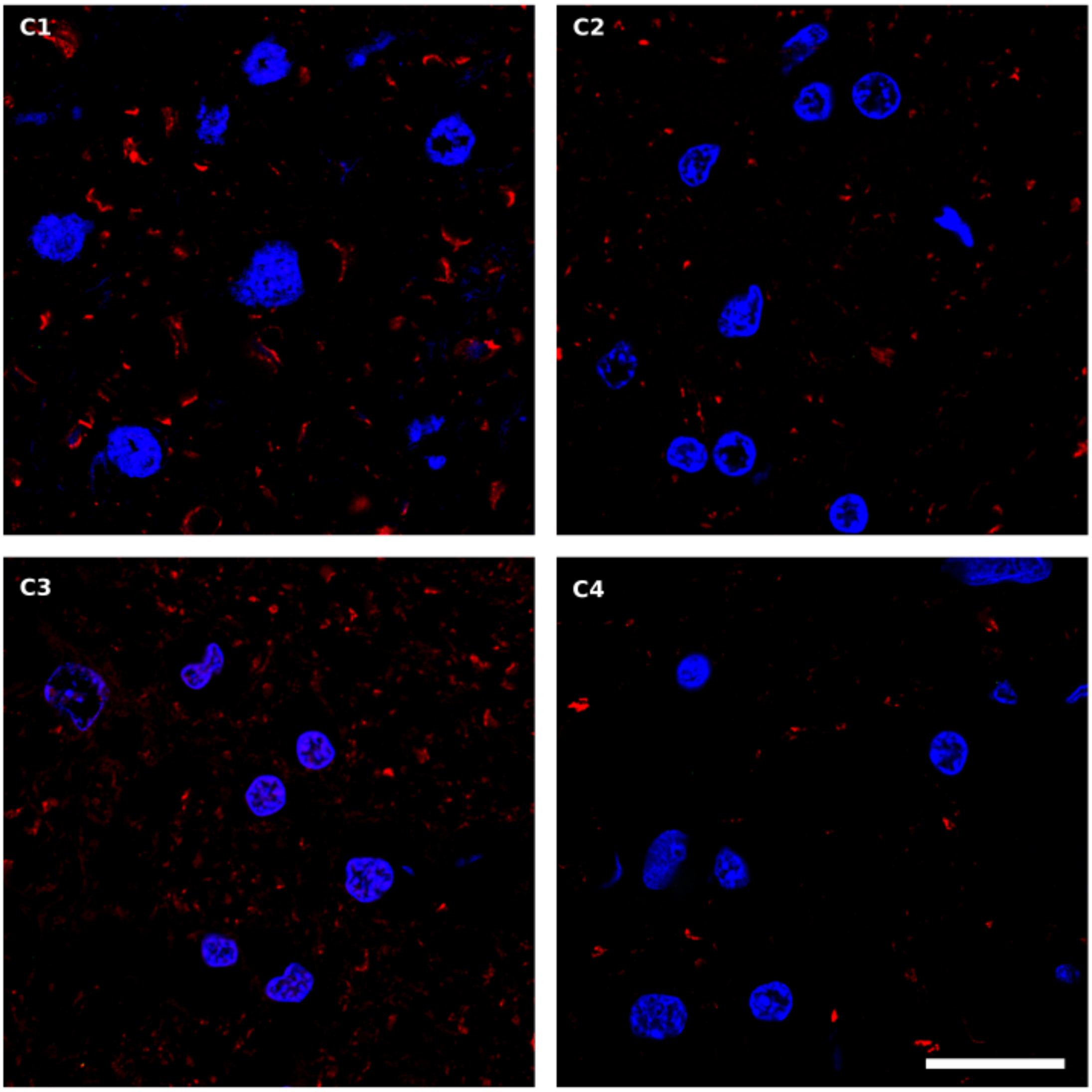

Supplementary Figure 5. Nested PCR analysis of fungal ITS1 and ITS2 in DNA extracted from frozen CNS tissue. Nested PCR of fungal DNA from PD patients. PCR analysis was carried out as described (see Materials and Methods). Agarose gel electrophoresis of the DNA fragments amplified by nested PCR. Panel A shows a schematic representation of fungal rRNA genes (18S, 5.8S and 28S rRNA) and the ITS1 and ITS2 regions, including location of the primers employed for the different nested PCRs; primers External 1 employed in the first PCR, primers Internal 1 employed in the second PCR to amplify ITS1, primers Internal 2 employed in the second PCR to amplify ITS2. Panel B shows nested PCR of ITS1, ITS2 in six PD patients. Upper panel, agarose gel electrophoresis of the DNA fragments amplified by nested PCR using DNA extracted from different regions of six PD patients using primers Internal 1 to amplify the ITS1 region. Bottom panel, agarose gel electrophoresis of the ITS2 region DNA fragments amplified from different CNS regions of six PD patients using primers Internal 2. C: Control PCR without DNA. CE: Control of DNA extraction without DNA. MC: motor cortex; MD: medulla; MS: mesencephalon; HT: hypothalamus; PN: pons; CB: callosal body; CLN: caudate and lenticular nuclei

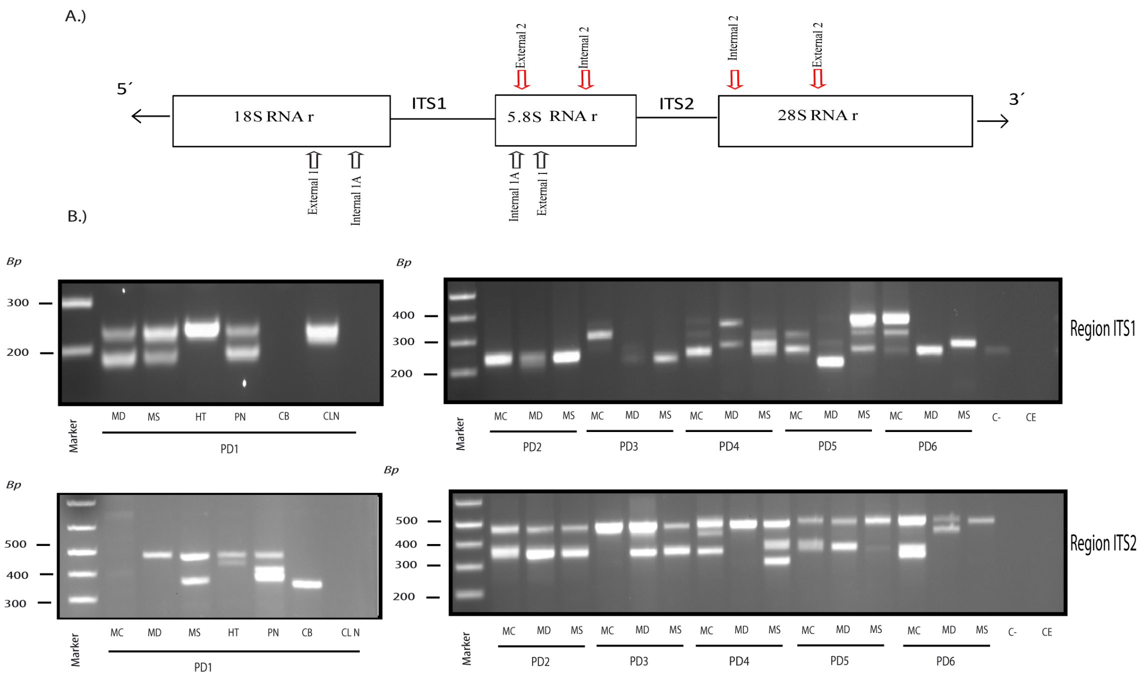

Supplementary Figure 6. Prokaryotic structures revealed by immunohistochemistry using an anti-*C. pneumoniae* antibody in CNS sections from PD patients. Three CNS regions (motor cortex, MC; medulla, MD; and mesencephalon, MS) of six PD patients were immunostained with a rabbit polyclonal antibody against *C. pneumoniae* (green) and a rat polyclonal antibody against *T. viride* (red). Nuclei were stained with DAPI (blue). Scale bar: 5 µm.

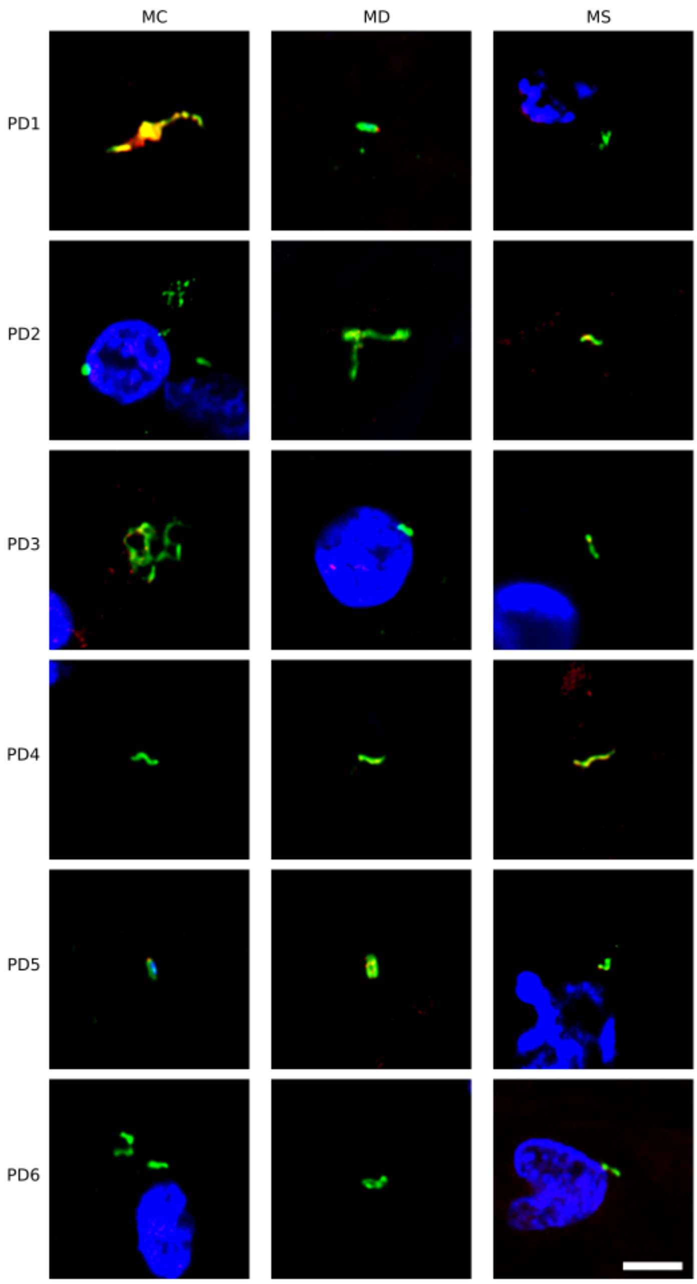

Supplementary Figure 7. Nested PCR analysis of the bacterial intergenic sequence region in DNA samples from frozen CNS tissue. PCR analysis was carried out as described in Materials and Methods. Panel A shows a schematic representation of the intergenic sequence (IGS) region, including the location of the primers employed for the different reactions. Panel B shows agarose gel electrophoresis of the DNA fragments amplified by nested PCR; analysis of different regions from six patients amplifying the IGS region. Primers 1406 (F)–559 (R) and 1492 (F)-242 (R) were used in the first and second round PCR, respectively: C: control PCR without DNA; CE: control of DNA extraction; MC: motor cortex; MD: medulla; MS: mesencephalon; HT: hypothalamus; PN: pons; CB: callosal body; CLN: caudate and lenticular nuclei.

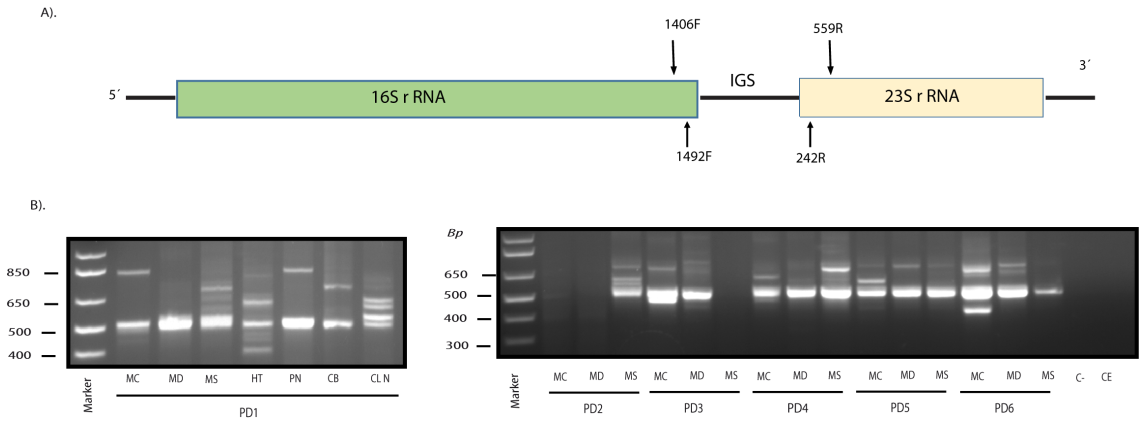

Supplementary Figure 8. Bacterial antigens in *corpora amylacea* observed in CNS sections from PD patients. Different sections from PD patients were incubated with a mouse monoclonal antibody against bacterial peptidoglycan (green), and a rabbit polyclonal antibody against *C. albicans* (red). Panels A–C: patient PD1; panels D and E: PD2; panels F and G: PD3; panels H and I: PD4; panels J–L: PD5; and panels M–O: PD6. Panels A, D, H, K and N: medulla; panels B, E, G, I, L and O: mesencephalon; panel C: callosal body; and panels F, J and M: motor cortex. DAPI appears in blue. Scale bar: 20 μm.

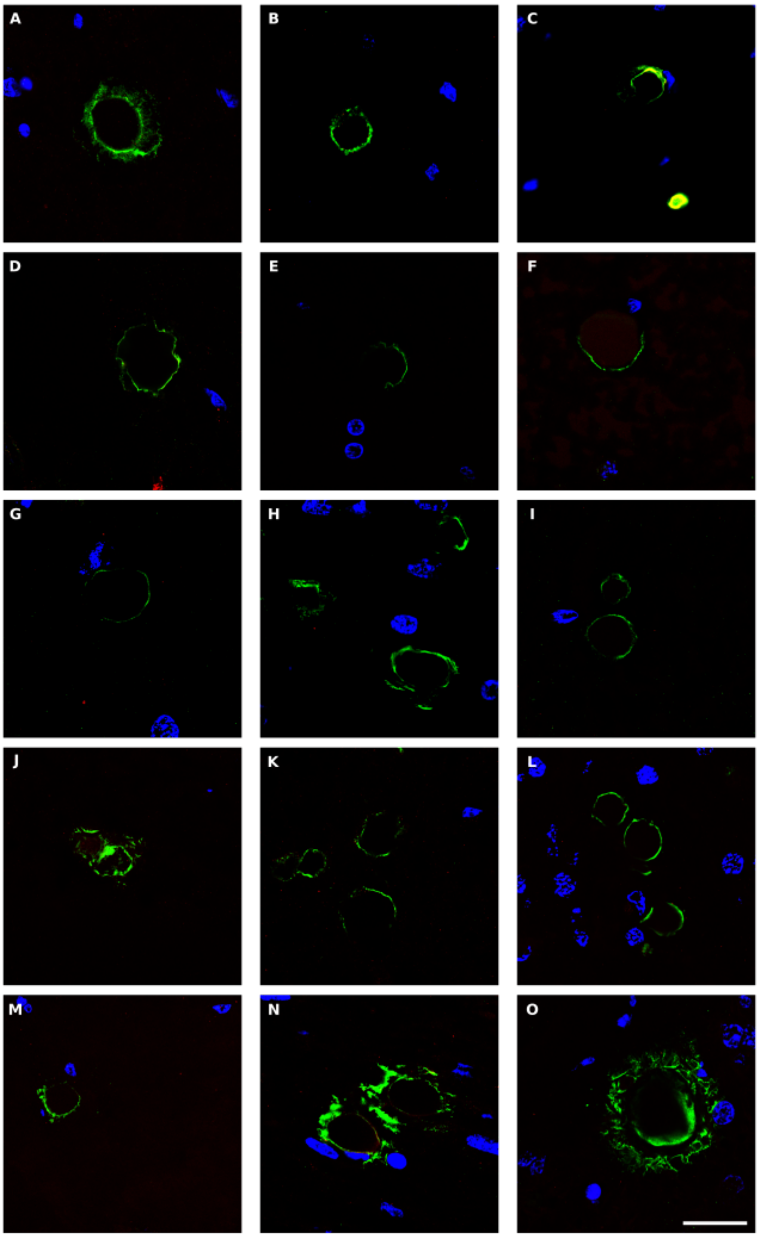

Supplementary Table I. Age and gender of PD patients studied in this work

PD patients	AGE	GENDER	CONTROLS	AGE	GENDER
PD1	65	MALE	C1	53	MALE
PD2	77	MALE	C2	78	MALE
PD3	84	MALE	C3	74	FEMALE
PD4	79	FEMALE	C4	83	FEMALE
PD5	82	MALE			
PD6	89	MALE			

Supplementary Table II. Fungal species identified by NGS

PD1 MC		*PD1 MD*		*PD1 MS*		*PD1 HT*		*PD1 PN*		*PD1 CB*		*PD1 CLN*	
Original paired reads	809499	Original paired reads	779383	Original paired reads :	760401	Original paired reads :	756460	Original paired reads :	757500	Original paired reads :	745017	Original paired reads :	696133
Joined sequences (%)	62,3	Joined sequences (%)	19,31	Joined sequences (%):	68,8	Joined sequences (%):	63,9	Joined sequences (%):	55,6	Joined sequences (%):	66,33	Joined sequences (%):	65,2
Uncultured candida	*18,7*	*Uncultured candida*	*20,7*	*Uncultured fungus clone S24T*	*4,4*	*Candida_sp*	*40,7*	*Xylaria_curta*	*21,9*	*Uncultured Ascomycota*	*47,5*	*Uncultured trichosporon*	*27,4*
Penicillium_digitatum	*7*	*Uncultured fungus clone S24T*	*4,5*	*Candida deformans*	*2,5*	*Xylaria_curta*	*16,6*	*Uncultured Trichosporon*	*14,9034661*	*Cryptococcus_curvatus*	*26,2*	*Candida_sp*	*21,7*
Uncultured fungus clone S24T	*4,1*	*Candida deformans*	*2,6*	*Uncultured fusarium*	*2,5*	*Cryptococcus_magnus*	*11,1*	*Uncultured candida*	*12,9*	*Cryptococcus_magnus*	*12,6*	*Xylaria_curta*	*19,2*
Xylaria_curta	*3,3*	*Uncultured fusarium*	*2,6*	*Uncultured malassezia*	*2,3*	*Uncultured candida*	*7*	*Cryptococcus_curvatus*	*8,8*	*Xylaria_curta*	*9,8*	*Uncultured basidiomycota*	*10,8*
Candida deformans	*2,3*	*Uncultured malassezia*	*2,4*	*Uncultured fungus clone S346*	*1,8*	*Uncultured basidiomycota*	*3*	*Uncultured basidiomycota*	*6,2*			*Uncultured candida*	*3,5*
Uncultured fusarium	*2,3*	*Uncultured fungus clone S346*	*1,9*	*Botrytis cinerea*	*1,8*	*Uncultured fungus clone S24T*	*1,5*	*Candida_sp*	*3,2*			*unculturedCladosporium*	*2,2*
Uncultured malassezia	*2,1*	*Botrytis cinerea*	*1,9*	*Malassezia globosa*	*1,5*			*Uncultured fungus clone S24T*	*2,8*			*Cryptococcus_curvatus*	*1,1*
Botrytis cinerea	*1,7*	*Malassezia globosa*	*1,6*	*Ascomycota sp*	*1,3*			*Candida deformans*	*1,6*				
Uncultured fungus clone S346	*1,7*	*Ascomycota sp*	*1,3*	*Uncultured fungus clone S44T_79*	*1*			*Uncultured fusarium*	*1,6*				
Ascomycota sp	*1,2*	*Uncultured fungus clone S44T_79*	*1,1*	*Uncultured fungus isolate F-RISA*	*1*			*Uncultured malassezia*	*1,5*				
		Uncultured fungus isolate F-RISA	*1,1*					*Botrytis cinerea*	*1,2*				
								Uncultured fungus clone S346	*1,2*				
								Malassezia globosa	*1*				

PD2 MC		*PD2MD*		*PD2MS*		*PD3 MC*		*PD3 MD*		*PD3 MS*	
Original paired reads	772545	Original paired reads :	905022	Original paired reads :	870008	Original paired reads :	775680	Original paired reads :	796106	Original paired reads :	625270
Joined sequences(%)	29,2	Joined sequences (%):	58,79	Joined sequences (%):	56,6	Joined sequences (%):	58,3	Joined sequences (%):	52,6	Joined sequences (%):	79,7
fungal_sp_FEF3	*16,1*	*Davidiella_tassiana*	*37,8*	*Xylaria_curta*	*29,2*	*Uncultured candida*	*23,7*	*Uncultured candida*	*23,6*	*Uncultured candida*	*17,5*
Uncultured candida	*14,2*	*Xylaria_curta*	*19,6*	*Cryptococcus_curvatus*	*16,4*	*uncultured Filobasidiales*	*10,1*	*Candida_versatilis*	*10,9*	*Uncultured basidiomycota*	*7,7*
Uncultured basidiomycota	*10,9*	*Sporobolomyces_coprosmae*	*8,5*	*trichoderma_sp*	*14,7*	*Uncultured fungus clone S24T*	*5,2*	*Uncultured fungus clone S24T*	*5,2*	*unculturedsoil_fungus*	*4,4*
Cryptococcus_sp	*10,2*	*Uncultured candida*	*6,5*	*uncultured Ascomycota*	*9,4*	*unculturedAscomycota*	*4,9*	*Candida deformans*	*2,9*	*Uncultured fungus clone S24T*	*3,8*
Uncultured fungus clone S24T	*3,1*	*Emericella_nidulans*	*1,8*	*Uncultured candida*	*8,3*	*Uncultured malassezia*	*3,7*	*Uncultured fusarium*	*2,9*	*Candida deformans*	*2,2*
Candida deformans	*1,8*	*Uncultured fungus clone S24T*	*1,4*	*Uncultured fungus clone S24T*	*1,8*	*Candida deformans*	*3*	*Uncultured Malassezia*	*2,7*	*Uncultured fusarium*	*2,2*
Uncultured fusarium	*1,8*			*uncultured malassezia*	*1*	*Uncultured fusarium*	*3*	*Botrytis cinerea*	*2,2*	*Uncultured malassezia*	*2*
Uncultured malassezia	*1,6*			*Candida deformans*	*1*	*unculturedsoil_fungus*	*2,7*	*Uncultured fungus clone S346*	*2,1*	*Uncultured fungus clone S346*	*1,6*
Botrytis cinerea	*1,3*			*Uncultured fusarium*	*1*	*Botrytis cinerea*	*2,4*	*trichoderma_sp*	*1,9*	*Botrytis cinerea*	*1,6*
Uncultured fungus clone S346	*1,3*					*Uncultured fungus clone S346*	*2,1*	*Malassezia globosa*	*1,7*	*Malassezia globosa*	*1,3*
Malassezia globosa	*1,1*					*Ascomycota sp*	*1,5*	*Ascomycota sp*	*1,5*	*Ascomycota sp*	*1,1*
						Uncultured fungus clone S44T_79	*1,2*	*Uncultured malassezia*	*1,5*		
						Uncultured fungus isolate F-RISA	*1,2*	*Davidiella_tassiana*	*1,3*		
						Malassezia globosa	*1,1*	*Uncultured fungus clone S44T_79*	*1,2*		
						Uncultured fungus clone B3_1883	*1*	*Uncultured fungus isolate F-RISA*	*1,2*		
						Uncultured fungus clone FITS_HBP1	*1*	*Uncultured basidiomycota*	*1,1*		
								Uncultured fungus clone B3_1883	*1*		
								Uncultured fungus clone FITS_HBP1	*1*		

PD4 MC		*PD4 MD **		*PD4 MS*		*PD5 MC*		*PD5 MD*		*PD5 MS*	
Original paired reads :	614956	Original paired reads :	640520	Original paired reads :	735236	Original paired reads :	683167	Original paired reads :	861501	Original paired reads :	681001
Joined sequences (%):	71,9	Joined sequences (%):	54,6	Joined sequences (%):	63,2	Joined sequences (%):	58,3	Joined sequences (%):	67,4	Joined sequences (%):	61,1
Uncultured candida	*20,2*	*Uncultured candida*	*2,207510431*	*Uncultured basidiomycota*	*19,1*	*Uncultured candida*	*17,8*	*Uncultured Trichosporon*	*12,8*	*Uncultured candida*	*19*
Uncultured basidiomycota	*9,2*	*Uncultured fungus clone S24T*	*0,5*	*Uncultured candida*	*18,5*	*Uncultured basidiomycota*	*10,4*	*Cryptococcus_magnus*	*9,8*	*Uncultured basidiomycota*	*14,5*
Uncultured fungus isolate F-RISA	*5,6*	*Uncultured fusarium*	*0,3*	*Penicillium_digitatum*	*11,5*	*Uncultured Trichosporon*	*4,3*	*Uncultured candida*	*8,6*	*Uncultured fungus clone S24T*	*4,2*
Uncultured fungus clone S24T	*4,4*	*Candida deformans*	*0,3*	*Emericella_nidulans*	*4,6*	*Uncultured fungus clone S24T*	*3,9*	*trichoderma_sp*	*6,8*	*Rhodotorula_mucilaginosa*	*3,9*
Davidiella_tassiana	*3,2*	*Uncultured fungus clone S346*	*0,2*	*Uncultured fungus clone S24T*	*4,1*	*Davidiella_tassiana*	*3,1*	*Davidiella_tassiana*	*3,7*	*trichoderma_sp*	*3,7*
Candida deformans	*2,5*	*Uncultured fungus clone S44T_79*	*0,1*	*Candida deformans*	*2,3*	*Xylaria_curta*	*2,4*	*Xylaria_curta*	*3,4*	*Candida deformans*	*2,4*
Uncultured fusarium	*2,5*	*Ascomnycota sp*	*0,1*	*Uncultured fusarium*	*2,3*	*Rhodotorula_mucilaginosa*	*2,4*	*Ustilago_bullata*	*2,3*	*Uncultured fusarium*	*2,4*
Botrytis cinerea	*1,9*	*Uncultured malassezia*	*0,1*	*Malassezia globosa*	*2,1*	*Candida deformans*	*2,2*	*Uncultured fungus clone S24T*	*1,9*	*Penicillium_aurantiogriseum*	*2,1*
Uncultured fungus clone S346	*1,8*			*Uncultured malassezia*	*2,1*	*Uncultured fusarium*	*2,2*	*Exophiala_sp_NH1238*	*1,4*	*Uncultured fungus clone S346*	*1,7*
Malassezia globosa	*1,5*			*Uncultured fungus clone S346*	*1,7*	*ascomycete_sp_IBWF79B_90A*	*2,2*	*Uncultured basidiomycota*	*1,4*	*Chytridiomycota_sp_Mori_T23*	*1,5*
Ascomycota sp	*1,3*			*Botrytis cinerea*	*1,7*	*Penicillium_aurantiogriseum*	*2*	*Candida deformans*	*1,1*	*Botrytis cinerea*	*1,3*
Uncultured malassezia	*1,3*			*Uncultured fungus isolate F-RISA*	*1,2*	*Uncultured malassezia*	*2*	*Uncultured fusarium*	*1,1*	*Ascomycota sp*	*1,2*
Uncultured fungus clone S44T_79	*1*			*Ustilago_hordei*	*1,2*	*Botrytis cinerea*	*1,7*			*Uncultured malassezia*	*2,2*
Uncultured fungus isolate F-RISA	*1*			*Ascomycota sp*	*1,2*	*Uncultured fungus clone S346*	*1,6*				
						Xylaria_cubensis	*1,5*				
						Malassezia globosa	*1,4*				
						trichoderma_sp	*1,1*				
						Ascomycota sp	*1,1*				

PD6 MC		*PD6 MD*		*PD6 MS*	
Original paired reads :	744266	Original paired reads :	667716	Original paired reads :	667716
Joined sequences (%):	69,6	Joined sequences (%):	43,4	Joined sequences (%):	43,8
Uncultured basidiomycota	*13,3*	*Uncultured candida*	*8,9*	*Penicillium_digitatum*	*10,7*
Uncultured candida	*13,19545537*	*Uncultured fungus clone S24T*	*1,9*	*Uncultured candida*	*8,3*
Uncultured fungus clone S24T	*2,9*	*Candida deformans*	*1,1*	*Candida_sp*	*7,6*
Candida deformans	*1,6*	*Uncultured fusarium*	*1,1*	*Uncultured basidiomycota*	*6,8*
Uncultured fusarium	*1,6*	*Uncultured malassezia*	*1*	*unculturedzygomycete*	*2,9*
Uncultured malassezia	*1,5*			*Uncultured fungus clone S24T*	*1,8*
Botrytis cinerea	*1,2*			*Uncultured malassezia*	*1*
Uncultured fungus clone S346	*1,2*			*Candida deformans*	*1*
Malassezia globosa	*1*			*Uncultured fusarium*	*1*

Supplementary Table III. Bacterial species identified by NGS

PD1 MC		PD1 MD		PD1 MS		PD1 PN		PD1 HT		PD1 CB		PD1 CLN	
Streptococcus	33	Pseudomonas viridiflava	63,7	Propionibacterium acnes	21,2	Lentzea albidocapillata	75	Actinomycetospora	21	Streptophyta	31,3	Megamonas	77
Gaiellaceae	13	Elizabethkingia meningoseptica	8,69	Corynebacterium	19,4	Sphingomonadales	18	Roseococcus	19	Acinetobacter schindleri	26,7	Scardovia	11
Virgisporangium ochraceum	11	Brevundimonas diminuta	6,78	Peptoniphilus	19,3	Lentzea;Other	2,3	Sphingomonas;s__	11	Arthrobacter;Other	15,4	Propionibacterium acnes	3,7
Arthrobacter	8,7	Pseudomonas	5,6	Aerococcaceae	8,8	Lentzea violacea	1,3	Megamonas	6	Acinetobacter lwoffii	7,81	Veillonellaceae	3,6
Actinomycetales	7,8	Pseudomonas pseudoalcaligenes	2,39	OD1	3,7			Pseudanabaenales	5,2	Chloroflexi	5,07	Firmicutes	1,4
Streptococcus	5	Escherichia coli	1,38	Methylobacterium;s__	2,63			TM7	4,4	Lactobacillus;Other	3,93		
Myxococcales	3,7	Enterobacter cloacae	1,15	Lysinibacillus boronitolerans	2,6			Hymenobacter;s__	4,1	mitochondria;Other	1,68		
Spirobacillales	3,2			Actinobacteria	2,1			Corynebacterium;s__kroppenstedtii	2,5	Leptotrichia;s__	1,49		
Actinobacteria;Other	2,2			Neisseriaceae	1,89			Intrasporangiaceae	2,4				
Actinobacteria;Other	1,9			Propionibacterium	1,27			Sphingomonas;Other	2,4				
Thermoleophilia;Other	1,8			Paucibacter	1,24			Xenococcaceae;Other	2,4				
Streptococcus minor	1,5							Arthrobacter;Other	2,3				
Solirubrobacterales	1,3							Comamonadaceae;Other	1,1				

PD2 MC		PD2 MD		PD2 MS		PD3MC		PD3 MD		PD3 MS	
Streptococcus	34	Ralstonia	26,1	Porphyromonas	43,3	Marinilabiaceae	17	Corynebacterium;s__kroppenstedtii	19,25	Veillonella dispar	19,35
Leptotrichia	6,9	Pseudomonas viridiflava	20,5	Actinomycetales	23,6	Corynebacterium;s__kroppenstedtii	13	Rhizobiales	11,6	Atopococcus abaci	18,32
Haemophilus parainfluenzae	6,3	Pseudomonas pseudoalcaligenes	20,4	Actinobacteria	9,88	Microbacteriaceae	6,9	OD1	6,618	OD1	12,68
Streptococcus	5,1	Streptococcus	9,25	Lentzea albidocapillata	6,9	Microbacterium	6,5	Flectobacillus	6,179	Actinomyces	8,815
Corynebacterium	5	Atopococcus abaci	5,99	TM7	3,81	Staphylococcus	4,8	Atopococcus abaci	5,164	Aerococcaceae	8,217
Veillonella dispar	4,5	Pseudomonas	4,21	Streptophyta	3,34	Comamonadaceae	4,6	Cytophagaceae	5,043	Porphyromonas	6,196
Actinobacteria	3,2	Sphingomonas	4,11	Streptococcus	2,95	Propionibacterium acnes	4,3	Bacillus foraminis	2,636	Corynebacterium	5,037
Fusobacterium	3,1	Kaistobacter	2,74	Bacteroidales	1,24	Flavobacterium	4,1	Staphylococcus	2,408	Comamonadaceae	4,932
Porphyromonas	3					Sulfuricurvum kujiense	4	Flavobacterium	1,85	C39	4,17
Gemellaceae	2,7					Sulfurospirillum	3,8	Arthrobacter psychrolactophilus	1,308	Actinobacteria;Other	1,717
Corynebacterium	2,5					Neisseriaceae	3,1	Actinobacteria;Other	1,137	Legionellales	1,57
Corynebacterium;s__kroppenstedtii	2,3					Geobacillus	2,9				
Ralstonia	1,8					ACK-M1	2,8				
Nesterenkonia	1,7					Bacteriovoracaceae	2				
Prevotella	1,3					OD1	1,9				
Streptococcus minor	1,3					Candidatus Aquiluna rubra	1,9				
Granulicatella	1					Oxalobacteraceae	1,6				
						Asticcacaulis	1,5				
						Actinobacteria;Other	1,4				
						Rothia mucilaginosa	1,2				

PD4 MC		PD4 MD		PD4 MS		PD5 MC		PD5 MD		PD5 MS	
Corynebacterium;s__	22	Arthrobacter;Other	45	OD1	20,2	Butyricimonas;s__	14	Acinetobacter schindleri	38	Acinetobacter lwoffii	53,73
Streptococcus;s__	15	Peptostreptococcus;s__	11,8	Staphylococcus	10,4	OD1	11	_KD8-87	10,85	Acinetobacter schindleri	26,49
Prevotella	7	Staphylococcus	9,21	Bdellovibrio bacteriovorus	6,17	Acinetobacter schindleri	9,8	Nocardioidaceae	9,196	Corynebacterium;s__kroppenstedtii	9,938
Propionibacterium acnes	6,8	Corynebacterium;s__kroppenstedtii	6,46	Corynebacterium;s__kroppenstedtii	4,64	Acinetobacter lwoffii	7,8	Staphylococcus	7,09	Ellin517	1,75
Prevotella	3,6	Rothia mucilaginosa	5,5	Hyphomicrobium;s__	3,94	Varibaculum;s__	7,7	OD1	5,191	Acinetobacter;Other	1,208
Proteobacteria	3,5	Selenomonas;s__	2,53	Acinetobacter schindleri	3,92	Wohlfahrtiimonas;s__	6,1	Acinetobacter johnsonii	2,725	Acinetobacter johnsonii	1,185
Corynebacterium;s__kroppenstedtii	3,4	Flavobacterium;s__	2,33	Exiguobacteraceae	3,81	Bacteria	4,1	Flavobacterium;s__	1,718		
Nocardioides	2,3	Flavobacterium;Other	2,14	GN02	3,2	Veillonella dispar	3,9	Flectobacillus;s__	1,653		
Actinobacteria;Other	2,3	Propionibacterium acnes	1,92	Parvimonas;s__	3,08	Microbacterium;s__	3,3	Sphingobacteriales	1,564		
Bacillus foraminis_	2,2	Arthrobacter psychrolactophilus	1,45	Proteobacteria	2,92	Candidatus Aquiluna rubra	2,5	Propionibacterium acnes	1,362		
Corynebacterium;Other	2,2	Streptophyta	1,14	Sphingomonas;s__	2,74	Microbacteriaceae;Other	2,5	Pseudonocardia;s__	1,318		
Nocardioidaceae	2	Microbacteriaceae;Other	0,71	Leptotrichiaceae	2,7	Acidimicrobiales	2,3	Actinomycetospora	1,292		
Streptococcus;Other	1,9			Bdellovibrio;s__	2,62	Tepidimonas;s__	2,2	Acinetobacter;Other	1,21		
Veillonella dispar	1,7			Aerococcaceae	2,19	OD1	2,1	Microbacterium;s__	1,145		
Haemophilus parainfluenzae	1,6			MIZ46	2,16	Corynebacterium;s__kroppenstedtii	2,1				
Candidatus Aquiluna rubra	1,6			Peptoniphilus;s__	2,05	Actinobacteria;Other	1,5				
Microbacterium;s__	1,5			Kaistobacter;s__	1,93	Gemellaceae	1,3				
Leptotrichia;s__	1,4			Bacteria	1,81	Flavobacterium;s__	1,1				

Microbacteriaceae;Other 1,2		Corynebacterium;s__ 1,73			
Actinomyces;s__ 1,1		Sphingomonadaceae 1,59			
OD1 1,1		Fusobacterium;s__ 1,42			
Sphingomonas;s__ 1,1		Hydrogenophilus;s__ 1,1			
		Arthrobacter;Other 1,1			
		OD1 1,1			
		Staphylococcus;Other 1,09			
		Prevotella 1,05			

PD6 MC		PD6 MD		PD6 MS	
OD1	48	Acinetobacter schindleri	28	OD1	22,2
Faecalibacterium prausnitzii	24	Propionibacterium acnes	23	Arthrobacter;Other	14,7
Megamonas	13	Sphingomonas;s__	11	Corynebacterium;s__	11,7
Stenotrophomonas;s__	3,6	Arthrobacter;Other	11	Acinetobacter lwoffii	11
EW055	1,7	Gaiellaceae	6,9	Flavobacterium;s__	10,2
Flavobacterium;s__	1,3	Corynebacterium;s__	6,6	Propionibacterium acnes	4,66
Bacteria	1,2	Actinobacteria;Other	2,4	Actinobacteria;Other	2,85
Faecalibacterium;s__	1	Streptococcus;s__	1,1	Nocardioidaceae	2,61
		Nocardioidaceae	0,4	Chitinophagaceae;Other	2,25
				Streptococcus;s__	1,95
				ACK-M1	1,91
				Bacteria	1,81
				OD1	1,67
